# Converting Food Waste into Value-Added Products: A Review on Current Technologies, Challenges, and Future Perspectives

**DOI:** 10.3390/foods15142577

**Published:** 2026-07-22

**Authors:** Antonietta Baiano

**Affiliations:** Dipartimento di Scienze Agrarie, Alimenti, Risorse Naturali e Ingegneria (DAFNE), Università degli Studi di Foggia, Via Napoli 25, 71122 Foggia, Italy; antonietta.baiano@unifg.it

**Keywords:** agri-food by-products, bioactive compounds, biorefinery, circular bioeconomy, functional ingredients, green extraction technologies, resource recovery, sustainability, technology readiness level, waste-to-value

## Abstract

Food waste has emerged as one of the most pressing global sustainability challenges because of its environmental, economic, and social implications. Nearly one-third of the food produced worldwide is lost or wasted each year, contributing to greenhouse gas emissions, depletion of natural resources, and increasing food insecurity. Advances in circular bioeconomy concepts and sustainable processing technologies have transformed food waste from an environmental liability into a valuable feedstock for producing biofuels, bioplastics, bioactive compounds, functional ingredients, prebiotics, and other high-value products. This review critically examines current strategies for converting food waste into value-added products, including green extraction technologies and biochemical, thermochemical, enzymatic, and microbial approaches. Attention is given to major agri-food by-products, such as fruit pomace, vegetable residues, oilseed meals, dairy by-products, and agro-industrial wastes. Emerging developments involving biorefinery concepts, artificial intelligence, digital biorefineries, synthetic biology, and carbon-neutral production systems are also discussed. Furthermore, the review highlights recent applications of waste-derived fibers, antioxidants, and polyphenols in functional foods, especially bakery products. Finally, key challenges related to feedstock heterogeneity, process scalability, regulatory frameworks, economic feasibility, and sustainability assessment are critically analyzed together with future research directions supporting the transition toward resilient circular bioeconomy systems.

## 1. Introduction

Food waste is one of the most pressing global sustainability challenges of the twenty-first century. Approximately 1.05 billion tons of food are wasted annually worldwide, accounting for 19% of all food produced for human consumption. Households account for approximately 60% of total food waste generation, followed by food service operations (28%) and retail sectors (12%) [1]. These are in addition to around 5.5 billion tons of agricultural waste [2]. The main sources of food waste can be classified into the following categories: plant-based residues (fruit pomaces, peels, seeds, vegetable trimmings); animal-based residues (dairy by-products, fish waste, meat processing by-products); and household and food-service waste. This massive generation of agri-food waste contributes to significant environmental consequences since traditional food waste disposal methods such as landfilling and incineration are associated with leachate generation and consequent soil and groundwater pollution as well as with the emission of greenhouse gases (methane, fossil CO_2_, nitrous oxide) and toxic compounds, water scarceness, degradation of soil, and diminishing biodiversity [3]. In addition, economic and social consequences such as monetary losses and food insecurity must be considered [4].

The development of sustainable food waste management strategies has therefore become a priority within the framework of the circular economy and the 17 sustainable development goals (SDGs) that the United Nations wants to achieve by 2030 [5]. From this perspective, recent research demonstrates that food waste is not merely a disposal problem but, in the perspective of reduce, reuse, and recycle, it becomes an important resource that can be upcycled to transform discarded agri-industrial by-products into high-value bioactive extracts to recover compounds that significantly benefit human health and industry [6]. Food waste valorization aligns strongly with SDG 2 (Zero Hunger), 12 (Responsible Consumption and Production), and 13 (Climate Action) [5]. In addition, it offers additional sources of income. Pursuing the production of bio-based goods from food waste can lead to the development of emerging markets whose potential benefits include attracting new investors and creating green jobs [7].

The growing interest in food waste valorization has led to the development of numerous approaches, including biochemical conversion, thermochemical treatment, fermentation, hydrothermal catalysis, and biorefinery integration [2,7]. Among them, green extraction technologies have attracted increasing attention since they offer environmentally friendly alternatives to conventional methods [8]. However, the industrial application of these techniques is still in progress due to a series of issues to be resolved which include, but are not limited to, heterogeneity of food waste; seasonal variability; logistics and collection; extraction costs; regulatory issues; food safety concerns; scalability; and consumer acceptance.

Although several recent reviews have provided valuable overviews of food waste valorization technologies, circular bioeconomy strategies, and biorefinery concepts [9,10,11,12,13,14,15,16], most studies focus on specific valorization routes, product categories, or technological platforms. A critical synthesis integrating food-derived bioactive ingredients, functional food applications, emerging processing technologies, and the associated technical, economic, and regulatory challenges remains limited.

Therefore, the aim of this review is to provide a comprehensive overview of current strategies for converting food waste into value-added products. Attention is devoted to biological, chemical, and thermochemical valorization pathways, the production of food ingredients and bioactive compounds, recent advances in biorefinery concepts, and the major challenges that must be addressed to facilitate industrial implementation within a circular economy framework.

## 2. Sources and Composition of Food Waste

As highlighted in the previous section, food waste originates throughout the whole food supply chain, including agricultural production, processing, retail, food services, and households. Food waste generally contains high levels of organic matter, particularly carbohydrates, proteins, lipids, and minerals, but also dietary fibers, minerals, and bioactive compounds (Table 1), making it a valuable feedstock for biorefinery and circular bioeconomy applications. However, the composition of food waste is highly heterogeneous and depends on sectors, commodities processed, origin, seasonality, geographical location, processing conditions, and dietary habits.

Fruit and vegetable wastes arise during harvesting, transportation, sales, processing, and consumption. Liu et al. describe these wastes as rich in water, soluble carbohydrates, fiber, minerals, vitamins, polyphenols, and other bioactive compounds [17]. Ben-Othman et al. [18] further report that fruit-processing wastes include pomace, consisting of pulp, skin, seeds, and stem, and that peels, skins, and other non-edible fruit parts can contain higher amounts of bioactive compounds than edible portions.

Wine and grape-processing residues are included among agri-food wastes rich in phenolic compounds. Mir-Cerdà et al. [20] identify winemaking wastes as important matrices for polyphenol recovery, including compounds such as proanthocyanins and resveratrol derivatives. Hrelia et al. [21] also report that grape-processing by-products, particularly skins and seeds, are rich in antioxidant compounds.

Olive oil by-products include olive-oil processing wastes and olive leaves. Mir-Cerdà et al. [20] identify olive-oil waste among the main agri-food residues investigated for phenolic recovery, including oleuropein.

Cereal and pulse processing generates by-products such as germ, bran, husks, stems, and leaves; Liu et al. [17] report that rice, sorghum, barley, wheat, millet, corn, and buckwheat are major cereal sources, while processing residues include germ and bran. The same authors report that barley processing by-products contain vitamin E, phytates, phenolics, and insoluble dietary fiber, while rice bran and rice husk can yield phenolic compounds after enzymatic hydrolysis.

Dairy wastes include milk-processing wastewater and whey. Liu et al. [17] report that dairy wastes contain proteins, fats, sugars, carbohydrates, lipids, and minerals, while cheese production generates whey as a major discarded stream. Papirio et al. [22] specifically identify cheese whey as a carbon-rich liquid effluent generated during milk processing.

Meat, poultry, and egg processing wastes include feathers, hair, skin, horns, hooves, soft tissue, deboning remnants, bones, blood residues, animal fat, detergent residues, proteins, and organic matter [17]. Seafood wastes include shrimp shells, crab shells, prawn waste, fish scales, and crustacean endoskeletons; Liu et al. [17] report that shells and scales are rich in proteins, chitin, and calcium carbonate. Yoha and Moses [19] also describe fish bone as a major fish-processing by-product composed of organic and inorganic materials.

Agri-food by-products can also be classified according to their suitability for material recovery. Valle et al. [23] report that agri-food waste is a source of lignin, cellulose, pectin, and starch, and can also be used as a substrate to produce chitosan, polyhydroxyalkanoates, and polylactic acid through microbial fermentation. Marotta et al. [24] classify biopolymer feedstocks as plant-, animal-, and microbial-derived wastes and by-products, emphasizing that their chemical–physical properties depend on the source material and extraction process.

## 3. Agri-Food Waste Valorization Strategies

Agri-food waste valorization shifts waste from an environmental burden into renewable resources through several techniques that are described below.

### 3.1. Extraction of Bioactive Compounds

High-value molecules (e.g., polyphenols, antioxidants, pectin) can be isolated from fruit peels, seeds, and leaves for use in the food, cosmetic, and pharmaceutical industries. The application of conventional extraction technologies (solid–liquid extraction, Soxhlet extraction, maceration, hydro-distillation, steam distillation, etc.) creates a significant environmental impact because they use solvents and/or are energy-consuming. For this reason, and because they have been extensively discussed in the scientific literature, they were excluded from the discussion in this review. Instead, so-called green and emerging technologies are gaining increasing attention.

The efficient recovery of bioactive compounds requires the application of appropriate pretreatments to agri-food waste. Because agri-food wastes generally have a high moisture content, they should be dried (through conventional drying or freeze-drying) and stored under controlled conditions to prevent degradation of target compounds. Then, they need to be grounded or milled to reduce the particle size, increase surface area and facilitate the contact with the extraction medium. Sometimes, enzymatic hydrolysis, with enzymes such as cellulases, hemicellulases, and pectinases, can be used to break down the cellular structure and release the target compounds [25].

### 3.2. Green Extraction Technologies and Emerging Technologies

Green technologies offer environmentally friendly alternatives to conventional valorization strategies since they can be used to recover valuable compounds from agri-food waste without relying on toxic solvents or energy-intensive operations. With respect to conventional technologies, these modern methodologies offer higher efficiency, reduced solvent usage, use of solvents (such as water, ethanol, supercritical CO_2_), shorter treatment times, and lower energy requirements. Furthermore, the solvents used—mainly water, ethanol, and supercritical CO_2_—are generally recognized as safe (GRAS) [8]. The term “emerging technologies” is generally used for technologies that have demonstrated promising laboratory or pilot-scale results but are not yet fully established at an industrial scale.

#### 3.2.1. Ultrasound-Assisted Extraction (UAE)

It employs ultrasound waves in the 20 kHz–100 MHz range to recover bioactive compounds. UAE can be efficiently used to extract molecules such as carotenoids, polyphenols, aromatic compounds, and polysaccharides from plant-derived by-products. UAE is based on acoustic cavitation, a physical phenomenon occurring when ultrasonic waves generate cycles of compression and rarefaction in the liquid. The resulting microbubbles first grow and then implode, releasing energy and enhancing solvent penetration and compound release. Successful UAE depends on optimizing parameters such as ultrasonic power, frequency, duty cycle, extraction time, temperature, solvent type, and solid-to-liquid ratio based on the target compounds and the material analyzed [25]. The UAE presents many advantages with respect to solvent extraction, supercritical extraction, and microwave-assisted extraction, including high extraction yield, low cost of operation, shorter time requirement, energy efficiency, retention of bioactivity in extracted components, and high level of automation and possibility of extensive applications [26]. However, scalability remains an important challenge [27]. An interesting example is the extraction of bioactive compounds from citrus by-products (consisting of peel, pulp, and seeds) generated by the citrus juice and essential oil processing industries. According to Ballistreri et al. [28], UAE was effective if the following conditions were applied: frequency, 24 kHz; constant agitation, 200 rpm; temperature lower than 50 °C; six consecutive sonication cycles of 10 min each; 1 kg of dried lemon by-products in 10 L of a 50:50 (*v*/*v*) water–ethanol solution. Compared with conventional extraction, UAE allowed increased recovery of total flavanones (+25.9%), hydroxycinnamic acids (+10.3%), and total polyphenols (+20.5%), as well as increased antioxidant activity (DPPH activity, +6.0%; ORAC values, +9.6%).

#### 3.2.2. Microwave-Assisted Extraction (MAE)

Microwave-assisted extraction (MAE) is an innovative and efficient extraction technology that uses non-ionizing electromagnetic waves within a frequency range of 300 MHz to 300 GHz. MAE can be used to extract phenolic compounds from fruit by-products, dried fruit shells, and grain residues, as well as the extraction of pectin from fruit peels. The extraction process using microwaves is based on the capacity of particles within a matrix to absorb electromagnetic waves. During a microwave treatment, energy transfer occurs through ionic conduction and dipole rotation, inducing heating of solvents and matrices and accelerating the extraction process. MAE parameters include solvent selection, solvent-to-solid ratio, temperature, processing time, irradiation power, and additional factors such as stirring rate and the characteristics of the sample matrix [29]. MAE offers several advantages such as enhanced heat and mass transfer, reduced working times, increased yield, reduced use of solvents, improved purity of the extracted compounds, and the possibility of scaling from laboratory size to pilot plant and commercial installations. However, disadvantages such as low feasibility for reaction monitoring and expensive equipment must be underlined [30]. MAE has been efficiently used to extract antioxidants from hazelnut by-products [31]. Concerning extraction from hazelnut meal, the highest total antioxidant capacity (TAC) of 0.0291 mmol Trolox/g dry matter was obtained under the following conditions: T = 100 °C; extraction time 19 min; solvent-to-solid ratio 23 mL/g dry matter; and ethanol ratio in water 90%. For the extraction of antioxidants from hazelnut skin, the maximum TAC (3.125 mmol Trolox/g dry matter) was achieved at a temperature of 100 °C, an extraction time of 30 min, a solvent-to-solid ratio of 10 mL/g dry matter, and an ethanol ratio in water of 55%.

#### 3.2.3. Pressurized Liquid Extraction (PLE)

Pressurized liquid extraction (PLE) employs solvents at elevated temperatures and pressures to maintain the solvent in the liquid state above its atmospheric boiling point. Under these conditions, solvent diffusivity increases while viscosity and surface tension decrease, enhancing mass transfer and improving extraction efficiency. Parameters such as solvent, temperature, pressure, and time of extraction must be carefully chosen to assure efficiency [32]. PLE has been successfully applied for the recovery of polyphenols, flavonoids, carotenoids, and other bioactive compounds from fruit pomace, grape marc, olive residues, and cereal by-products. An example is represented by the optimized extraction of ellagitannins extracted from pomegranate skin under the following conditions: temperature, 200 °C; time, 20 min; solvent, 77% ethanol; pressure, 103 bar [33].

#### 3.2.4. Accelerated Solvent Extraction (ASE)

It uses moderate-to-high temperature and pressure to extract target compounds. With respect to traditional extractions that typically require 8 to 10 h, ASE reduces processing times from hours to 15–30 min and up to tenfold less organic solvent. In these conditions, efficiency increases since solvent diffusivity increases, viscosity reduces, and solute–matrix interactions weaken. The parameters to optimize include temperature, pressure, static cycle duration, and solvent polarity. ASE is highly selective, thus avoiding downstream purification requirements [34]. This technique can be effectively applied to the extraction of compounds such as phenolic acids, flavonoids, and other bioactive metabolites [35]. An interesting application of ASE is the obtainment of phenolic-rich extracts from apple peels [34]. Under optimized extraction conditions (108.5 °C, 2.03% ethanol concentration, 26 min extraction time), ASE extracts showed total phenolic content like those of conventional extracts but significantly higher than extracts obtained through sonication. Antioxidant activity values were comparable for ASE and UAE extracts, both higher than the conventional ones. In contrast, the highest total flavonoids were in the UAE extract, followed by ASE and conventional ones.

#### 3.2.5. Supercritical Fluid Extraction (SFE)

SFE is based on the use of a solvent in its supercritical state. At these conditions, the solvent behavior is between liquid and gas, which determines a higher diffusivity and solvating capacity compared to the liquid state. Carbon dioxide is the most used because of characteristics such as its chemical stability and the absence of toxicity and flammability; the obtainment of solvent-free extracts; the capability to extract non-polar and low-polar compounds; the possibility to modify its selectivity by combining it with an organic solvent; and finally, its critical state (31.1 °C and 7.3 MPa, respectively) that is easily achievable [29,36]. SFE is advantageous from the perspective of an industrial process, since it allows shortening of processing times, thus reducing energy consumption and increasing productivity. The main disadvantage is the influence of the matrix on process effectiveness, which requires careful optimization studies. SFE has been successfully applied to the extraction of carotenoids contained in waste deriving from carotenoid-rich fruits and vegetables (sweet potato, red tomato, yellow, red, and green bell pepper, pumpkin, peach, apricot) [37]. The total carotenoid recovery was greater than 90% *w*/*w* under optimized conditions (59 °C, 30 min; 350 bar, 15 g/min CO_2_, 15.5% (*v*/*v*) ethanol as co-solvent).

#### 3.2.6. Subcritical Water Extraction (SWE)

Water is a polar solvent and cannot be used to extract non-polar compounds that, instead, can be extracted with the often toxic, non-polar solvents. However, water polarity can be modified by adjusting pressure and temperature to extract compounds of different polarities. Water maintained between 100 °C and 374 °C and between 1 MPa and 22.1 MPa is in its subcritical state. In these conditions, water is in the liquid state. Other factors affecting the efficiency of SWE include particle size, solvent flow rate, and the addition of co-solvents. The advantages of using SWE also include low cost, possibility of avoiding the matrix drying stages, reproducibility, and scalability, while degradation of heat-sensitive compounds can be counted among disadvantages [38]. Compounds such as phenolic compounds, terpenes, flavonoids, anthocyanins, polysaccharides, amino acids and proteins, biopolymers, tannins, and fibers can be extracted and hydrolyzed with SWE [39]. Optimization of SWE conditions in terms of temperature, time, and flow rate depends on sources, target polymers, and desired extract composition [40].

#### 3.2.7. Hydrodynamic Cavitation (HC)

HC is an emerging non-thermal technology that produces cavitation phenomena. Liquid turbulence and bubble collapse facilitate the disruption of plant tissues, thus enhancing the release of matrix-bound compounds and the consequent mass transfer. HC is a continuous and easily scalable technology, characteristics that make it suitable for industrial applications concerning the extraction of bioactive compounds from waste and by-products [41]. As an example, HC has been efficiently applied to extract and recover phenolic compounds from [28]. The extraction was conducted through a centrifugal cavitator system, according to the following conditions: flow rate of 0.92 L/min; temperature below 50 °C; six consecutive cavitation cycles of 10 min each; 1.5 kg of dried lemon by-products in 15 L of a 50:50 (*v*/*v*) water–ethanol solution. Under these conditions, HC increased antioxidant activity (DPPH, +11.4%; and ORAC, +2.0%) and recovery of flavanone (+12.0%), hydroxycinnamic acids (+7.2%), and total polyphenols (+5.2%) with respect to UAE (results reported in the section dedicated to this technique).

#### 3.2.8. Cold Plasma-Assisted Extraction (CPAE)

Cold plasma is a non-thermal technology operating at near-room temperatures in which ionized gases generate reactive species. Gases can be converted in plasma (the fourth state of matter) by supplying a high amount of energy. The ionized gas contains atoms, electrons, free radicals, gas molecules, photons, and ions in balanced concentration so that plasma is electrically neutral. The presence of charged particles allows plasma to conduct electricity. In cold plasma, the electrons lie at a higher temperature than the heavy particles (gas and ions), which are at room temperature. Reactive species such as ozone and hydroxyl radicals, which interact with substrates, inducing chemical modifications and enhancing extraction efficiency. This is why high-energy electrons and ions disrupt cellular matrices, thereby releasing bioactive compounds while preserving thermolabile nutrients. CPAE is often used in combination with other technologies. As an example, it has been combined with UAE to extract phenolic compounds from spent coffee grounds [42]. Advantages and disadvantages of CPAE are highly interconnected. Scalability, economic viability and extraction efficiency can be high, but they require a fine optimization of CPAE treatment parameters (duration, plasma-source-to-sample distance, sample layer thickness) [42]. Moreover, high-fat matrices suffer from plasma-induced lipid oxidation unless noble gases are used. However, noble gases entail high procurement costs and require high-voltage equipment and stringent safety protocol [43].

#### 3.2.9. Ohmic Heating-Assisted Extraction (OHAE)

OHAE relies on the application of electric fields. Briefly, the matrix to heating is crossed by an alternating electric flow, and heat is generated first by the migration of ions in an electrolyte from an electrode toward an oppositely charged one and then by the resistance derived from the collisions between ions. Electric current and heat promote the release of molecules of interest from the matrix. Target compounds that can be extracted by application of OHAE comprise phenolics, essential oils, pectins, proteins, and cellulose fibers [44]. Heating speed and uniformity of OHAE allow time savings and reduction in energy consumption and avoid the degradation of heat-sensitive compounds. However, OEAE is not suitable for matrices having low electric conductivity unless a pretreatment for the incorporation of salts and minerals or the reduction in particle size is done. In the same way, low-conductivity solvents can potentially reduce extraction yield. An interesting application of OHAE is the extraction of pectin from grapefruit, lemon, and orange waste at 80 °C, at processing times from 0 to 180 min. The EC value was measured between 1.46 and 2.06 S/m during heating. The total energy consumption was noted to rise with extended processing times. This process involved extended treatment at specific pH and yielded from 9 to 18% pectin depending on processing time and type of matrix [45].

#### 3.2.10. Membrane Separation Technologies

The application of membrane-based pressure-driven processes for the separation and recovery of compounds from dilute streams shows the following advantages over traditional separation methods: selectivity, low operating temperature (20–60 °C), low energy requirements, no need for the use of chemicals, low space requirement, possibility of automation, minimization of water consumption (thanks to recycling of process water), easy scalability, and integration with other separation techniques. Separation efficiency depends on membrane material, pore size/molecular weight cut-off, and operating conditions such as transmembrane pressure, feed concentration, flow rate, pH, and temperature. The separation mechanisms operating in the various types of filtrations are the following: size exclusion (microfiltration, MF; ultrafiltration, UF; nanofiltration, NF); macromolecular shape (UF); steric hindrance (NF); and solution diffusion (RO) [46]. As an example of membrane technology application, volatile fatty acids have been efficiently separated from ultrafiltered effluent rich in these compounds by nanofiltration through thin polypiperazine membranes at temperatures of 20–21 °C and applied pressure of 15 bars [47].

#### 3.2.11. Electrodialysis

Electrodialysis is a separation process in which electrically charged membranes, and the application of an electrical potential difference are used to separate ionic species from a solution and other uncharged compounds [48]. A recent application of this technique concerned the purification of an antihypertensive chicken by-product hydrolysate fraction. Electrodialysis with ultrafiltration membranes allowed a peptide-selective separation based on their charge and molecular weight [49].

#### 3.2.12. Pulsed Electric Field (PEF) Extraction

PEF is based on the application of short pulses of high electric fields (10–80 kV/cm) and durations from microseconds to milliseconds in a product located in a PEF chamber between electrodes. Being a non-thermal technology, it is effective in eliminating microorganisms and enzymes while preserving the nutritious value of a food. These electrical pulses can produce temporary pores in cell membranes, allowing ions and molecules to flow in and out of the cell. By compromising cell structural integrity, intracellular molecules may leak out of the cell. PEF can be applied to the extraction of compounds such as carotenoids, polyphenols, ascorbic acid, and hesperidin [50]. A comparison among applications of PEF and other techniques (conventional, UAE, and high hydrostatic pressure) to the extraction of flavonoids from orange flavedo and albedo was performed by Afifi et al. [51]. The author observed that the optimum processing conditions for albedo and flavedo extraction were 15 kJ/kg at 10 kV and 15 kJ/kg/3 kV, respectively.

#### 3.2.13. High Hydrostatic Pressure (HHP) Extraction

HHP is a non-thermal method usually performed in the 100–1200 MPa pressure range. The high pressure applied determines increased plant cell permeability, leading to increased diffusivity and solubility of compounds. HHP has been efficiently used in the recovery of pectin from fruit waste [52]. As an example, the extraction of flavonoids from orange flavedo and albedo has been successfully obtained by applying HHP at 200 and 400 MPa, respectively [51].

#### 3.2.14. Deep Eutectic Solvents (DES), Natural Deep Eutectic Solvents (NADES), and Pressurized Natural Deep Eutectic Solvents (p-NADES)

They are an emerging class of sustainable solvents that is attracting research due to their unique properties. DES are made of a hydrogen bond acceptor (e.g., quaternary ammonium salts) and one or more hydrogen bond donors (e.g., amines, carboxylic acids), which together lower the mixture’s melting point. These solvents are GRAS, chemically and thermally stable, non-flammable, cost-effective, easy to use, and biodegradable. However, the main limit to their application is the difficulty in separating them from the reaction products. They have been widely used for the extraction of hydrophilic molecules (phenolic compounds, anthocyanins) but also for the extraction of non-polar compounds (carotenoids) [8,53,54]. Deep eutectic solvents have been applied for the recovery of phenolics from barley malt rootlets. The use of choline chloride-malic acid (1:2 molar ratio) as a deep eutectic solvent guaranteed a maximum extraction of 9.51 ± 0.83 gallic acid equivalents/g of barley malt rootlets under the following optimal extraction conditions: 1:21 solid-to-liquid ratio, 80 °C, 43 min, and 29% as a percentage of water in the deep eutectic solvent [55].

### 3.3. Biochemical Conversion

Biochemical conversion technologies employ microorganisms and enzymes to transform food waste into valuable products.

#### 3.3.1. Anaerobic Digestion (AD)

Anaerobic digestion is one of the most mature and widely implemented technologies for the valorization of agri-food waste, enabling the conversion of organic residues into renewable energy and nutrient-rich by-products. The process is based on the microbial degradation of organic matter in the absence of oxygen through a sequence of hydrolysis, acidogenesis, acetogenesis, and methanogenesis reactions, resulting in the production of biogas, mainly composed of methane and carbon dioxide, and a stabilized digestate that can be further utilized as an organic fertilizer or soil amendment. Due to its operational flexibility, high technology readiness level (TRL 9), and ability to simultaneously recover energy and nutrients, AD is currently considered one of the most effective strategies for integrating food waste management within a circular bioeconomy framework. Food waste represents an excellent substrate for AD because of its high biodegradability and elevated content of carbohydrates, lipids, and proteins. Nevertheless, its heterogeneous composition, seasonal variability, and susceptibility to acidification may negatively affect process stability and methane yields. Consequently, considerable research has focused on pretreatment technologies and co-digestion strategies to improve substrate biodegradability and optimize reactor performance. A representative example is provided by the co-digestion of cheese whey and industrial hemp hurds investigated by Papirio et al. [22]. Cheese whey, a highly biodegradable dairy by-product characterized by elevated organic content, produced a biochemical methane potential of ~446 mL CH_4_/g volatile solids, whereas hemp hurds, a lignocellulosic agricultural residue, yielded around 242 mL CH_4_/g volatile solids. When both substrates were combined at a cheese whey-hemp hurds ratio equal to 70:30, methane production increased by 10.7% compared with the theoretical value calculated from the individual substrates, demonstrating a clear synergistic effect of co-digestion. The authors further estimated that biomethane production from these agri-food residues could generate net profits of up to €6124/ha while contributing to the decarbonization of the transportation sector through the production of renewable biomethane.

Recent developments in AD have moved beyond the traditional objective of biogas generation toward the concept of integrated anaerobic digestion biorefineries. It should represent the central platform of a circular valorization strategy integrating pretreatment technologies, advanced reactor configurations, biogas upgrading systems, and digestate valorization pathways [56]. Furthermore, the digestate generated during AD can be processed through mechanical, physicochemical, or biological treatments to recover nitrogen, phosphorus, and organic matter, thereby closing nutrient cycles and reducing reliance on synthetic fertilizers.

#### 3.3.2. Fermentation

Fermentation is increasingly recognized as one of the most versatile and sustainable approaches for the valorization of agri-food waste and by-products, owing to its ability to convert low-value biomass into a broad spectrum of high-value compounds (bioactive compounds, functional ingredients, organic acids, enzymes, single-cell proteins, biofuels, and biopolymers) for food, feed, pharmaceutical, cosmetic, and chemical applications through the metabolic activity of microorganisms [57].

Among fermentation processes, a fundamental distinction depends on oxygen management. Aerobic pathways generally allow both a faster microbial growth and a more efficient energy yield. Anaerobic processes are often characterized by slower metabolic rates, but they are easier to scale in industrial settings as they eliminate the high energy costs and technical complexities associated with oxygen supply [58]. Industrial fermentation systems can be further divided into submerged fermentation (SmF) and solid-state fermentation (SSF), depending on the physical nature of the substrate and operational strategy. In SmF, microorganisms grow in a liquid medium. However, the high energy requirement due to both the mechanical stirring necessary to keep the medium homogeneous and the possible air injection remains a major challenge for the scalability of these systems [59].

The mostly employed microorganisms include lactic acid bacteria (LAB), yeasts, filamentous fungi, and *Bacillus* species, each characterized by distinct metabolic capabilities. LAB are used to produce lactic acid, bioactive peptides, vitamins, and exopolysaccharides, while simultaneously improving the nutritional quality and digestibility of agri-food residues [60]. Filamentous fungi, particularly *Aspergillus niger* and *Aspergillus oryzae*, have demonstrated remarkable potential to produce hydrolytic enzymes [cellulases, xylanases, tannases, and β-glucosidases], which facilitate the release of bound phenolics from plant cell walls of grape pomace, avocado seeds, pineapple residues, and barley bran [61,62,63,64].

Another rapidly growing application is the production of single-cell proteins, which are increasingly regarded as sustainable alternatives to conventional protein sources [36]. Fruit and vegetable wastes, olive residues, brewer’s spent grain, and food waste hydrolysates have been utilized as fermentation substrates for yeasts such as *Saccharomyces cerevisiae*, *Candida utilis*, and *Yarrowia lipolytica*, resulting in protein-rich microbial biomass containing up to 40–45% protein on a dry-weight basis [65,66,67]. In addition, lactic acid bacteria, *Bacillus* spp., and filamentous fungi have been employed to hydrolyze proteins present in whey, soybean residues, coffee grounds, fish wastes, and cereal by-products, producing peptides with antioxidant, antihypertensive, antimicrobial, and health-promoting activities [68,69,70].

Beyond food and nutraceutical applications, fermentation technologies have also been applied to the production of industrially relevant compounds such as citric acid, lactic acid, polyunsaturated fatty acids, pigments, flavor compounds, bioplastics, and biofuels [57].

#### 3.3.3. Enzyme-Assisted Extraction (EAE)

Enzymatic approaches provide environmentally friendly alternatives for extracting valuable compounds from food waste. Enzymatic-assisted extraction is widely used to valorize agri-food waste because it enables the recovery of valuable compounds (polyphenols, flavonoids, carotenoids, pectins, proteins, dietary fibers, oils). In matrices such as fruit peels, pomace, seeds, bran, and vegetable residues, those compounds are often trapped within plant cell walls. Enzymes such as cellulases, pectinases, hemicellulases, and proteases break down structural components of the biomass, releasing more target compounds and improving extraction yields. Because EAE generally works under mild conditions (lower temperatures, controlled pH, and short processing times), the extracted compounds retain higher biological activity and quality. However, enzymes are very expensive, and their use needs setting enzymes’ optimum performance conditions, thus restricting their industrial applications [71]. Mazzocchi et al. [72] applied an enzyme mix (cellulase, 40%; xylanase, 41%; and polygalacturonase, 19%) to extract food-grade chlorophyll-based green colorant from unsold spinach. The conditions able to maximize the amount of chlorophyll include a temperature of 25 °C; a time lower than 2 h; a zinc concentration of 150 ppm; and an enzyme mix dose between 12 and 45 U/g.

### 3.4. Thermochemical Conversion

Thermochemical methods involve high-temperature processing to produce energy and carbon-rich materials.

#### 3.4.1. Pyrolysis and Gasification

Pyrolysis converts food waste into biochar, biooil, and syngas under oxygen-limited conditions. It has gained the attention of researchers due to its operational simplicity and adaptability to several feedstocks. The major limitation of applying pyrolysis to agri-food waste is its high moisture, which requires a lot of energy for preliminary operations such as drying and grinding, thus leading to high greenhouse gas emissions. However, integrated pyrolysis processes use non-condensable gases or biochar generated during pyrolysis to dry food waste or supply heat for reactor operation. In this way, such a closed-loop approach can contribute to the process’s self-efficiency as well as to the carbon-neutral potential since produced carbon dioxide can re-enter the photosynthetic cycle, while biochar soil application promotes long-term carbon sequestration [73,74,75].

As an example of application, starting from a waste blend of rice and French fries, a high char yield (212 g/kg) can be obtained through pyrolysis performed at 650 °C together with an amount of captured or removed CO_2_ from the air equal to 536 g/kg of food waste [76]. Biochar derived from food waste exhibits high adsorption capacity and environmental remediation potential towards Pb^2+^ and phenols [77].

#### 3.4.2. Hydrothermal Carbonization (HTC)

Hydrothermal carbonization transforms highly wet biomass (water content, 60–90%) into hydrochar—a carbon-rich and energy-dense material like lignite coal—under moderate temperatures (180–260 °C) and pressure (35–55 bar). HTC is particularly suitable for high-moisture food waste. The hydrothermal carbonization process includes the following steps: hydrolysis, dehydration, polymerization, and carbonization [78,79]. The conversion of this technology to an industrial-scale system has already been successfully tested in pilot plants and full-scale facilities in Europe and China [80].

### 3.5. Insect-Assisted Pretreatment

Saprophagous insects have gained attention as pretreatment agents for transforming organic waste, reducing its heterogeneity, and generating nutrient-rich biomass suitable for further valorization. Among insects, the black soldier fly (*Hermetia illucens*) has been extensively studied for its ability to convert high-moisture waste such as manure [81]. In more depth, black soldier fly larvae (BSFL) can rapidly reduce substrate mass, convert nitrogen-rich waste into protein-rich biomass, and modify microbial community [82]. BSFL followed by *Protaetia brevitarsis* larvae (PBL) can produce a two-stage insect-assisted system capable of performing a chicken manure humification. BSFL treatment results in rapid detoxification, strong ammonification, and organic matter and total nitrogen reduction. A net increase in total humus and humic acid is then registered after the PBL stage [83].

### 3.6. Direct Composting

Direct composting is the traditional and most widespread method for nutrient recycling, in which biodegradable waste is broken down by bacteria, actinomycetes, and fungi to create agricultural amendments and nutrient-rich fertilizers. Microbial degradation can occur through a faster aerobic pathway or a slower anaerobic pathway. To avoid excessive greenhouse gas formation, especially in anaerobic composting, and pathogen development, microbial inoculum can be considered. The ideal moisture content for composting should be in the 40–65% range. If it is higher, porosity and oxygen flow decrease. If it is lower, microbial growth and metabolism are impaired [84,85,86].

Table 2 summarizes key agricultural by-products, the methods used to process them, and their resulting value-added products, while Table 3 describes representative applications of green and emerging extraction technologies.

### 3.7. Biorefinery, Artificial Intelligence and Digital Biorefineries

**Figure 1:**
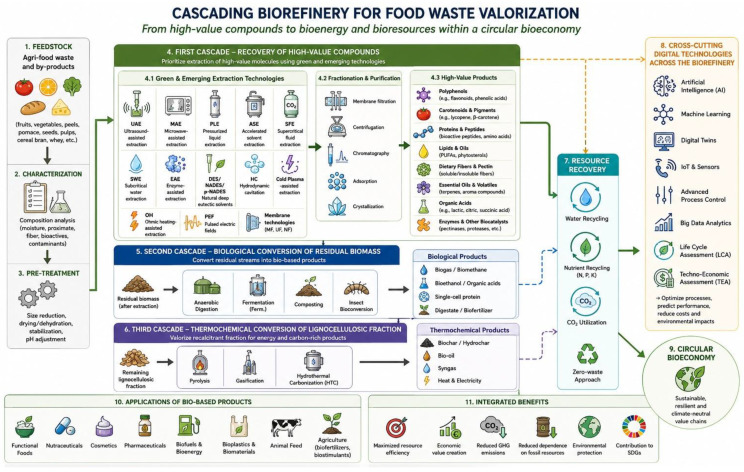
Conceptual framework of an integrated cascading biorefinery for the valorization of agri-food waste and by-products. The figure illustrates the sequential recovery of high-value compounds through green extraction technologies, followed by biological and thermochemical conversion of residual biomass, resource recovery, and integration of digital technologies within a circular bioeconomy. The figure was developed based on concepts described in the cited literature.

The concept of a biorefinery has emerged as a key pillar of the bioeconomy and circular economy, aiming to maximize the value obtained from renewable biological resources, since it offers an integrated framework for converting agri-food waste into multiple value-added products, rather than targeting a single output. As in the case of a petroleum refinery, a biorefinery integrates a range of conversion technologies to transform biomass into multiple marketable products and energy carriers, thus emphasizing resource efficiency [120,121,122]. Furthermore, in this model, heterogeneous residues from agricultural production and food processing—such as peels, pomace, husks, bagasse, whey, oilseed cakes, cereal residues, and vegetable by-products—can be treated. One of the most widely accepted definitions was developed by the International Energy Agency (IEA) Bioenergy Task 42, which defines biorefining as the “sustainable processing of biomass into a spectrum of marketable products and energy” [120]. Figure 1 shows the conceptual framework of an integrated cascading biorefinery for the valorization of agri-food waste and by-products. This definition was successfully expanded to emphasize the production of a portfolio of bio-based products, including food and feed ingredients, chemicals, materials, minerals, and CO_2_-derived products, together with bioenergy in the form of fuels, power, and heat [121]. From a technological perspective, biorefineries integrate physical, chemical, thermochemical, and biochemical conversion processes to fractionate biomass and valorize its major components, such as carbohydrates, proteins, lipids, and lignin, into high-value products [122,123]. The objective is not only the generation of biofuels but also the co-production of food ingredients, feed, biomaterials, and biochemicals, thereby improving resource efficiency and economic viability [122,124]. Cherubini [124] described the biorefinery as the sustainable equivalent of the petrochemical refinery, replacing fossil resources with biomass to produce energy and chemicals. Consequently, modern biorefineries are considered essential infrastructures for the transition toward sustainable food systems, circular bio-economies, and reduced dependence on fossil resources [121,124].

Several case studies demonstrate that agri-food waste biorefineries are most effective when designed according to a cascade logic, in which high-value compounds are recovered first, and residual fractions are subsequently converted into energy, fertilizers, or platform chemicals.

A representative example is grape pomace, one of the main by-products of winemaking. A multi-step cascading biorefinery has been proposed for red grape pomace, combining recovery of polyphenols by supercritical CO_2_ extraction with subsequent production of volatile fatty acids and other products from residual biomass. This approach shows how winery residues can be upgraded into nutraceutical ingredients, microbial substrates, and bioenergy carriers rather than being used only as compost or animal feed [125]. Orange peel waste is another well-studied feedstock. A lab-scale biorefinery loop for orange peels involving limonene recovery, volatile fatty acid production, and activated carbon generation was developed by Rizzioli et al. [126]. Solid/liquid extraction of limonene was performed with n-hexane at 85 °C with an orange peel-to-solvent ratio of 2:1, obtaining a limonene recovery yield of 1.20% *w*/*w*. After that, a volatile fatty acid yield of about 43% was realized through a hydraulic retention time of 5 days and a total solid inlet content of 10% *w*/*w*. At the end, the materials were first converted into biochar through slow pyrolysis at 550 °C for 1 h and then physically activated with CO_2_ at 880 °C for 1 h. A further example concerns tomato pomace (tomato peels and seeds), generated by the canning industry, which is used to produce biogas. A tomato pomace biorefinery based on supercritical CO_2_ extraction was developed by Scaglia et al. to recover lycopene, followed by valorization of the remaining biomass for bioenergy and digestate production [127]. Authors found that lycopene recoveries depended on the changes induced in peel structure by the pre-extraction drying step. High moisture values permitted high lycopene recovery (around 97%) and gave a water-in-oil emulsion as an extract. After oil extraction, exhaust tomato peels are highly biodegradable. This strategy is attractive because tomato pomace is rich in carotenoids but is commonly destined to low value uses such as animal feed or anaerobic digestion. Contrarily to the actual use, supercritical CO_2_ extraction combined with anaerobic digestion can determine an economic revenue equal to € 787.9/t. Cheese whey represents a highly relevant case for dairy biorefineries. Recent studies describe whey as a lactose-, protein-, and mineral-rich stream that can be fractionated into whey protein concentrates and then fermented into bioethanol, lactic acid, or other biochemicals. A process simulation was designed for an industrial-scale facility located in Apulia (Italy) to treat 539 m^3^/day of an industrial-scale biorefinery model designed for Apulia—processing 539 m^3^/day of raw cheese whey. It integrated three cascade processes: membrane filtration to recover 56% *w*/*w* whey protein concentrate; subsequent fermentation of lactose by *Kluyveromyces marxianus* to produce fuel-grade ethanol; and anaerobic digestion of residual sludge for combined heat and power generation from cheese whey. An integrated cheese whey biorefinery combining protein recovery, ethanol fermentation, anaerobic treatment, energy generation, and soil conditioner production was proposed by Colacicco et al. [128]. Olive oil production wastes are also promising biorefinery feedstocks. Studies on olive mill wastewater and olive pomace have proposed integrated schemes for recovering phenolic compounds, producing biogas or biomethane, and generating biofertilizers. Serrano et al. [129] designed a high-temperature thermal pretreatment of olive pomace carried out at 170 °C for 60 min and a subsequent phenol extraction (yield of 1600 mg hydroxytyrosol per 1 kg of substrate). A further anaerobic digestion step was used to stabilize the pre-treated olive mill solid waste and to recover methane. Anaerobic bioreactors for biogas production were designed by Montegiove et al. [130] to valorize the residual of the protein hydrolysis process of three-phase olive pomace and the waste recovered after the extraction of bioactive molecules from olive mill wastewater. These approaches are particularly relevant for Mediterranean countries, where olive residues are abundant and seasonally concentrated.

Digitalization is increasingly recognized as a key enabler of sustainable biomass valorization and circular bioeconomy systems. Recent studies highlight that the integration of artificial intelligence (AI), big data analytics, Internet of Things (IoT) technologies, blockchain, and digital twins can improve resource efficiency, process optimization, traceability, and decision-making throughout biomass value chains and biorefinery operations [131,132]. Digital tools are particularly relevant in biomass-based systems because they facilitate the management of large and heterogeneous datasets generated during biomass production, feedstock characterization, processing, logistics, and product distribution [131].

AI and machine learning (ML) have emerged as powerful tools for predictive modeling and process optimization. Machine learning algorithms such as Random Forest, Support Vector Machines, Artificial Neural Networks, and Deep Learning models have demonstrated high accuracy in predicting biomass yields, crop productivity, and biomass quality from climatic, agronomic, and remote-sensing data [131]. In addition, AI models have been successfully applied to optimize thermochemical conversion processes, including biomass gasification, enabling accurate prediction of gas composition and energy performance while reducing experimental requirements [131]. These capabilities can support more efficient feedstock selection and process design in future food waste biorefineries.

A digital twin (DT) represents another emerging component of smart biorefineries. By integrating real-time process data with virtual process models, digital twins enable continuous monitoring, predictive maintenance, scenario simulation, and dynamic process optimization [133]. Digital twins can reduce scale-up risks by continuously reconciling model predictions with plant data, facilitating the early detection of performance drift and improving process robustness. Their integration with AI-driven predictive models further supports adaptive process control and operational optimization in complex biomass conversion systems.

The role of digital technologies extends beyond process optimization. Blockchain systems have been proposed to enhance traceability and transparency throughout biomass and food waste supply chains, while IoT-enabled sensor networks allow real-time monitoring of material and energy flows [131]. Similarly, digital technologies can facilitate the implementation of circular economy principles by improving waste tracking, predictive waste analytics, logistics optimization, and resource recovery strategies across agri-food systems [132].

Artificial neural network modeling has been used to optimize extraction of exosomes for cosmetic applications from purslane. Data concerning extraction parameters such as temperature, solid–liquid ratio, duration, and solvent type as well as the concentrations of the corresponding flavonoid extracts have been recovered from the literature and entered in the Pythia program. Data were modeled through the so-called ‘Evolutionary Optimization’ future function to predict potential outcomes for new input values, eliminating the need for further experiments [134].

The great amount of agricultural and municipal organic solid wastes produced annually (1300 million tons and 2.01 billion tons, respectively) provides feedstock for biomass briquettes [135]. Since quality parameters (calorific value, density, compressive strength, and durability) of briquettes influence combustion efficiency, they need to be evaluated through time-consuming, labor-intensive, and expensive destructive testing [136]. Machine learning enables the analysis of large datasets in real time by leveraging historical data and integrating multiple variables, providing fast, non-destructive, and accurate predictions of briquette properties [137].

A digital twin is very useful for maintaining biorefinery efficiency. A DT is a virtual representation of a real entity. Digital and real entities are connected to each other, sharing real-time functional and operational data to increase biorefinery efficiency. A case study of Jankovich et al. [138] describes the design and implementation of a digital twin to produce synthetic natural gas from biogenic feedstock and improve efficiency by up to 5% thanks to the fully autonomous plant operation that requires only minimal supervision. A conceptual design of a biorefinery based on microalgae biomass feedstock with the final output of methanol production is described in the work of Moretta et al. [139]. The methanol production was modeled with PythonTM (v3.9), while process simulations were computed using Aspen HYSIS^®^ v11, an industrial simulation package of the state of the art.

### 3.8. Hybrid Extraction Systems

The strategies described above can be used in combination to obtain synergistic results. Some examples are described below.

#### 3.8.1. DES and MAE

DESs were coupled with MAE to extract active compounds from cocoa bean shells, and the results were compared to those obtained from DES extraction alone [140]. When the two technologies were applied, theobromine and caffeine yields were in the 2.502–5.004 mg/g and 0.778–1.599 mg/g ranges, respectively. When DES was applied as a standalone technology, the corresponding ranges were 2.145–4.682 mg/g and 0.681–1.524 mg/g, respectively.

#### 3.8.2. SPE and SWE

A recent study describes the combination of PEF and SWE to extract hesperidin and narirutin from Satsuma mandarin (*Citrus unshiu*) peels. The application of PEF at 3 kV/cm for 120 s followed by SWE at 150 °C for 15 min resulted in an increase in hesperidin and narirutin extraction yields by 22.1 and 33.6% compared to the application of SWE alone [141].

#### 3.8.3. AD and Gasification

AD and gasification technologies show limitations such as incomplete biomass conversion and excessive digestate production. Hybrid systems combining the two strategies enable the use of diverse biomass (municipal solid waste, agri-food waste), and they are highly efficient in converting feedstocks into renewable energy (result attributed to the effective conversion of cellulose-rich stover) and valuable products like biochar, particularly if they use heat from gasification to dry AD residues [142]. From an economic point of view, hybrid AD-gasification systems reduce the need for larger digester sizes, reducing the amount of invested capital and increasing ROI [143]. Challenges concern scalability of hybrid systems, management of feedstock variability, balancing of AD, and gasification processes. By providing real-time monitoring and predictive analysis, digital twins could increase both the efficiency and economic viability of these hybrid systems [144]. From an environmental point of view, hybrid AD and gasification systems reduce GHG emissions with respect to the standalone techniques since AD reduces methane emissions by converting organic waste into biogas, while gasification minimizes CO_2_ emissions through both energy conversion processes and carbon sequestration in biochar [142].

### 3.9. Technology Readiness Level (TRL) Analysis

While numerous technologies have been proposed for food waste valorization, their maturity and commercial applicability vary considerably. Therefore, assessing the technology readiness level (TRL) is essential for identifying technologies that are likely to be implemented at an industrial scale in the short and medium term. The TRL framework, originally developed by NASA and subsequently adopted by the European Commission, classifies technologies on a scale from 1 (basic principles observed) to 9 (full commercial deployment), providing a useful tool for evaluating technological maturity and supporting decision-making processes in biorefinery development.

Among the available valorization technologies, anaerobic digestion represents one of the most mature solutions, with a TRL of 9 and widespread commercial implementation worldwide to produce biogas and biomethane from food waste and agri-industrial residues [22,56]. Conventional fermentation processes can also be considered highly mature technologies, generally ranging from TRL 8 to 9, owing to their extensive industrial application [57,60].

Ultrasound-assisted extraction, microwave-assisted extraction, and supercritical fluid are currently positioned at TRLs between 6 and 8, with numerous pilot-scale and industrial applications already reported for the recovery of polyphenols, carotenoids, and other bioactive compounds from agri-food by-products [29,32]. Conversely, emerging technologies such as deep eutectic solvents, natural deep eutectic solvents, microfluidic extraction systems, and hybrid extraction platforms generally remain at lower TRLs (3–5), as further optimization and scale-up studies are still required before commercial deployment [57].

Novel valorization pathways including insect bioconversion, single-cell protein production from food waste, and food-waste-derived bioplastics occupy intermediate positions, typically between TRL 5 and 8, reflecting the increasing number of pilot and demonstration-scale facilities currently operating worldwide [86,88]. In contrast, digital biorefineries, artificial intelligence-assisted process optimization, and digital twin technologies remain at relatively low maturity levels (TRL 2–5), despite their significant potential to improve process efficiency, resource utilization, and economic performance in future circular bioeconomy systems [57].

## 4. Production of Value-Added Products from Food Waste

**Figure 2:**
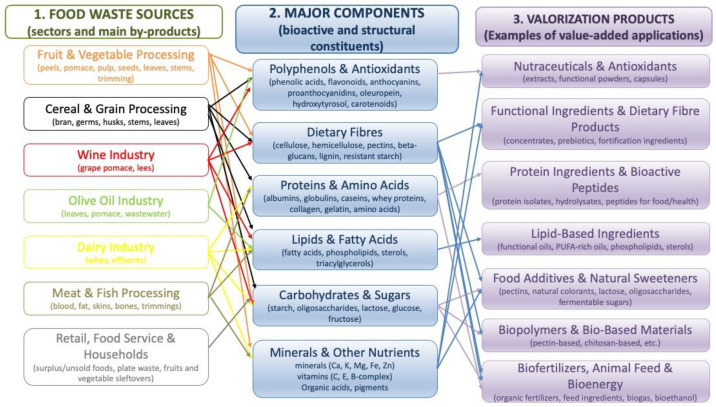
Valorization pathways of the different food waste sources within a circular bioeconomy framework.

Food waste valorization can generate a remarkably broad range of value-added products (Figure 2), which can be classified according to their market sector.

### 4.1. Food Ingredients and Nutraceuticals

The recovery of functional ingredients and nutraceutical compounds is among the most economically attractive valorization pathways. Fruit and vegetable by-products, grape pomace, olive residues, cereal bran, and dairy by-products contain substantial quantities of dietary fibers, proteins, peptides, polyphenols, carotenoids, vitamins, phytosterols, and other molecules with recognized health-promoting properties [18,20]. Even food waste is often richer in bioactive compounds than edible portions (think, for example, of the phenolic content of vegetation water compared to extra virgin olive oil).

Dietary fibers recovered from fruit pomace, cereal bran, and vegetable residues are increasingly incorporated into food formulations to improve nutritional quality and support gastrointestinal health. Similarly, protein concentrates and bioactive peptides derived from whey, fish-processing residues, and brewer’s spent grain have attracted considerable interest as functional food ingredients [18]. Polyphenol-rich extracts obtained from grape pomace, olive leaves, citrus peels, and other plant-derived wastes are widely investigated for their antioxidant, anti-inflammatory, and antimicrobial activities and are commonly formulated as nutraceutical supplements, functional powders, capsules, and tablets [21,145].

### 4.2. Natural Food Additives

Food waste also represents an important source of natural additives that can replace synthetic ingredients. The increasing consumer demand for clean-label products has further stimulated interest in waste-derived natural additives, creating new market opportunities for agri-food by-products.

Pectin extracted from citrus peels and apple pomace is widely used as a gelling, stabilizing, and thickening agent in food products. Likewise, natural pigments such as anthocyanins from grape pomace and carotenoids from tomato by-products can be used as natural colorants. Essential oils recovered from citrus residues exhibit antimicrobial and antioxidant properties and have been proposed as natural preservatives for food applications [18,145].

### 4.3. Biopolymers and Sustainable Biomaterials

The production of biopolymers and biodegradable materials is one of the fastest-growing sectors in food waste valorization. Agri-food residues contain natural polymers such as cellulose, hemicellulose, lignin, starch, pectin, and proteins that can be directly used or further processed into sustainable materials [23]. Moreover, food waste can serve as a substrate for the microbial production of biopolymers such as polyhydroxyalkanoates (PHAs) and polylactic acid (PLA), which are increasingly used for biodegradable packaging applications [88]. Other examples include pectin-based films, cellulose-derived materials, chitosan coatings, and lignin-based composites. These biomaterials offer environmentally friendly alternatives to petroleum-derived plastics and contribute to reducing the environmental impact of food packaging systems [24].

### 4.4. Agricultural Products: Biofertilizers, Bio-Stimulants, and Biopesticides

Agricultural applications represent another important valorization route. Organic residues rich in nutrients and bioactive compounds can be converted into biofertilizers, bio-stimulants, and biopesticides that improve crop productivity while reducing dependence on synthetic agrochemicals [89,90].

Biofertilizers produced from food waste contribute to nutrient recycling by returning nitrogen, phosphorus, potassium, and micronutrients to agricultural soils. Bio-stimulants derived from plant extracts, protein hydrolysates, and phenolic-rich fractions have been shown to enhance plant growth, nutrient uptake, and stress tolerance. In addition, several food-processing residues contain natural antimicrobial and insecticidal compounds that can be exploited in the formulation of biopesticides [90].

### 4.5. Chemicals and Industrial Products

Food waste biorefineries can also generate chemicals that can be used in the manufacture of solvents, polymers, and specialty chemicals. These products obtained from renewable waste streams are increasingly considered a key strategy for reducing dependence on fossil-based resources and promoting sustainable industrial development. As examples, compounds such as lactic acid, succinic acid, citric acid, acetic acid, levulinic acid, furfural, and hydroxymethylfurfural can be produced through fermentation, hydrolysis, and catalytic conversion processes [146].

### 4.6. Animal Feed Ingredients

Food waste and processing by-products have long been utilized as feed ingredients, and this remains one of the most common valorization pathways since it contributes to resource efficiency by reducing competition for conventional feed resources while simultaneously minimizing waste disposal. Fruit pomace, vegetable residues, brewer’s spent grain, whey, and other nutrient-rich by-products can be incorporated into animal diets after appropriate treatment and stabilization [17].

### 4.7. Bioenergy and Biofuels

At the end of the biorefinery cascade or when higher-value recovery options are not feasible, food waste can be converted into renewable energy and biofuels. Anaerobic digestion is one of the most widely applied technologies, producing biogas and biomethane from food-processing residues, municipal food waste, and agricultural by-products [22]. Other energy products obtained from food waste include bioethanol, biodiesel, biohydrogen, biochar, and solid biofuels [146]. Although energy recovery generally generates lower economic returns than nutraceutical or functional ingredient production, it remains an important component of integrated biorefinery systems because it enables the utilization of residual biomass that cannot be efficiently valorized through other pathways.

### 4.8. Cosmetic and Pharmaceutical Applications

Many compounds recovered from food waste exhibit biological activities that make them attractive for cosmetic and pharmaceutical applications. Polyphenols, carotenoids, essential oils, antioxidant peptides, collagen, and gelatine are increasingly incorporated into skincare products, anti-aging formulations, wound-healing materials, and pharmaceutical preparations [18]. Particularly promising are grape pomace, olive by-products, citrus residues, fish skins, and fish bones, which provide valuable bioactive molecules with antioxidant, antimicrobial, anti-inflammatory, and tissue-regenerating properties.

## 5. Overview of Commercial Products Currently on the Market That Use Upcycled Ingredients

The commercialization of upcycled foods has expanded rapidly during the last decade, transforming food upcycling from an emerging sustainability concept into a recognized market segment. A major driver of this growth has been the development of the Upcycled Certified^®^ certification program established by the Upcycled Food Association (UFA), which provides a standardized framework for verifying that ingredients and products utilize materials that otherwise would not have gone to human consumption and are sourced through traceable supply chains with measurable environmental benefits [147]. Briefly, the Upcycled Certified scheme is the first third-party certification for ingredients/foods made using surplus food or by-products. Possession of this certification allows producers to apply a recognized mark on the packaging of certified products, proving to consumers that their purchase actively mitigates food waste. To obtain certification, a product must meet three requirements: it must contain ingredients that otherwise would not have gone to human consumption; ingredients must come from a verifiable and auditable supply chain; and a positive environmental impact must be obtained. Upcycled Certified^®^ has the following three upcycled input thresholds for certification:

- Upcycled Ingredient (UI): ≥95% upcycled input(s) by weight (excluding added water)
- Product Containing Upcycled Ingredients (PUIs): ≥10% upcycled input(s) by weight (excluding added water)
- Minimal Content: <10% upcycled input(s) by weight (excluding added water).

The global market of upcycled food products is around USD 41.71 billion in 2025. It is expected to grow from USD 44.68 billion in 2026 to USD 79.51 billion by 2034, at a CAGR of 7.47%. North America holds a market share of 55.74% in 2025, followed by Europe (22.55%) and the Asia-Pacific area (17.90%). Compared to other certification programs, upcycled certification is considered the fastest-growing standard scheme. According to “an important third-party food verification firm in the U.S.”, 568 upcycled products were certified in 2024, with an increase of 17% with respect to the previous year [148]. Based on the distribution channel, the supermarkets/hypermarkets segment will represent 48.84% market share in 2026, being able to provide a wide range of upcycled ingredients/foods in various pack sizes [148]. The future market trends can be summarized as follows [149]:

- Application of koji fermentation to upcycle vegetable waste/spent brewer’s grains into sauces and condiments;
- Side streams become mainstream products. An example is represented by oat protein from oat milk production, now reconsidered as high-protein new bakery flours;
- Fruit pulp and discarded juice from juicing operations are mixed to create new natural functional beverages;
- Increasing cross-industry application, with agricultural leftovers used to produce pet foods and personal care products.

Commercial products currently available on the market (Table 4) can be grouped into several categories according to the type of upcycled ingredient utilized and the final product application.

### 5.1. Bakery Products and Upcycled Flours

One of the most mature market segments involves the recovery of plant-based processing by-products for flour production.

Renewal Mill is a US producer that commercializes, Organic Okara Flour, produced from the fiber-rich pulp remaining after soymilk production, together with other upcycled ingredients including oat protein, pineapple fiber, green banana flour, and white corn flour [150]. These ingredients are currently incorporated into cookies, baking mixes, crackers, and other bakery products.

Similarly, ReGrained (Berkeley, CA, USA) developed SuperGrain+^®^ flour, an ingredient derived from brewer’s spent grain generated during beer production. SuperGrain+^®^ is currently utilized in snack bars, cereals, bakery products, pasta, and plant-based foods [151]. Notably, SuperGrain+^®^ became the first ingredient to receive Upcycled Certified^®^ status from the UFA [147].

### 5.2. Snack Foods

They represent one of the fastest-growing categories of upcycled foods.

Pulp Pantry (Los Angeles, CA, USA) produces vegetable chips manufactured from vegetable pulp remaining after cold-pressed juice extraction. The company also incorporates upcycled okara flour into several formulations to improve nutritional value and fiber content [152].

Rind Snacks (New York City, NY, USA) commercializes dried fruit snacks containing edible peels that are typically removed during conventional fruit processing. Current product lines include dried oranges, kiwifruit, apples, and tropical fruit blends [153].

Barnana (Santa Monica, CA, USA) utilizes surplus and cosmetically imperfect bananas that do not meet retail specifications to produce banana chips, bites, and plantain-based snacks [154].

### 5.3. Beverages

The beverage sector provides several examples of food upcycling.

Toast Brewing (London, UK) produces beer using surplus bread recovered from bakeries and food-service operations. Bread partially replaces malted barley during brewing, allowing the recovery of bakery products that would otherwise be wasted [155].

Take Two Foods (Portland, OR, USA) commercializes barley-based milk alternatives produced from spent barley recovered from brewing operations [157].

### 5.4. Functional Ingredients

This sector currently represents one of the largest commercial opportunities for food waste valorization.

EverGrain^®^ (AB InBev, St. Louis, MI, USA) produces protein and fiber ingredients recovered from brewer’s spent grain. These ingredients are supplied to food manufacturers for incorporation into beverages, plant-based foods, nutritional products, and dietary supplements [156].

Renewal Mill supplies a portfolio of upcycled ingredients, including okara flour, oat protein, pineapple fiber, green banana flour, and white corn flour, for use in bakery products, snacks, and functional foods [150].

Upcycled Foods, Inc. (formerly ReGrained) has expanded beyond spent grain ingredients and currently develops fruit-derived ingredients, syrups, cocoa substitutes, teas, and purées obtained from side streams of food manufacturing processes [151].

### 5.5. Ready-to-Eat Foods and Sauces

Matriark Foods (New York City, NY, USA) develops sauces, soups, broths, and culinary bases produced from surplus vegetables and processing by-products recovered from farms and food manufacturers. These products are marketed both to consumers and institutional food-service operators [158].

### 5.6. Premium Specialty Foods

Upcycled ingredients are increasingly incorporated into premium food categories.

Salt & Straw (Portland, OR, USA) has launched limited-edition product collections (premium ice cream formulations) utilizing ingredients such as okara, spent grain, surplus bread, and cacao fruit pulp [159].

## 6. Circular Economy and Sustainability Perspectives of Converting Food Waste into Value-Added Products

**Figure 3:**
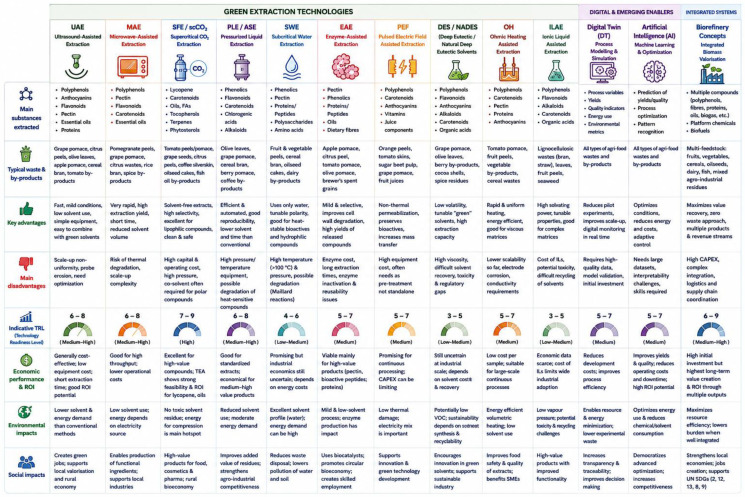
Comparison of some green extraction technologies for agri-food waste valorization according to technological, economic, environmental, and social performance.

Food waste valorization strongly supports the transition toward circular bioeconomy systems, which became a central objective of global sustainability policies, including the European Green Deal, the Circular Economy Action Plan, and the Farm-to-Fork Strategy. Within this framework, food waste is increasingly recognized as a secondary resource capable of generating environmental, economic, and social value through integrated valorization pathways. The transition from a linear to a circular food system is increasingly recognized as essential for achieving sustainable development goals because it is intended to maintain the value of resources for as long as possible through reuse, recycling, recovery, and regeneration processes. Recent studies indicate that integrated biorefinery systems can simultaneously contribute to environmental protection, economic growth, and social well-being, thereby supporting the already mentioned multiple SDGs [10,91]. Environmental, economic and social sustainability should be assessed with caution, because food waste valorization does not automatically generate benefits. Figure 3 offers a comprehensive comparison of several green extraction technologies for agri-food waste valorization according to technological, economic, environmental, and social performance.

### 6.1. Environmental Sustainability

The environmental benefits of food waste valorization arise primarily from the reduction in landfill disposal, greenhouse gas emissions, and resource depletion. The recovery of valuable compounds from agri-food residues also reduces the demand for virgin raw materials and promotes more efficient resource utilization.

A notable example is the valorization of olive mill wastewater, one of the most environmentally problematic residues generated by the olive oil industry. Due to its high organic load and phenolic content, untreated olive mill wastewater can cause severe soil and water contamination. The review of Roig et al. [160] highlighted that integrated treatment and valorization strategies can transform olive mill waste into a source of antioxidants, biofertilizers, and bioenergy while significantly reducing its environmental impact. In more depth, as most olive oil producer countries in the Mediterranean area are exposed to desertification processes, the use of olive mill wastes as soil conditioners or fertilizers would be beneficial to improve the soil fertility and control the erosion processes, while in organic agriculture, the use of organic matter from olive mill waste could close the cycle of residues–resources.

One of the most thoroughly investigated case studies concerns brewer’s spent grain (BSG), the major by-product of the brewing industry. Petit et al. [161] performed a detailed environmental assessment of different preservation and stabilization pathways for BSG intended for human food applications. In more depth, they conducted a comparative study of the environmental performance of different stabilization strategies (dehydration, separation, lactofermentation, freeze-drying, refrigeration, freezing, methanation) with respect to composting and use for animal feed. In the current state of research, innovative scenarios have been evaluated as having more impact than conventional ones because they all involved energy-consuming technologies and involved transportation logistics, emphasizing the importance of process optimization for maximizing sustainability benefits.

Another relevant example is represented by apple pomace, one of the most abundant by-products generated by the fruit juice industry. A recent consequential Life Cycle Assessment (LCA) conducted in Germany evaluated the environmental implications of diverting apple pomace from conventional uses toward black soldier fly larvae production. The study demonstrated that valorization pathways can generate significant environmental benefits when by-products substitute conventional feed ingredients and when regional logistics are considered [162]. In more depth, diverting the apple pomace from its current utilization as biogas substrate or ruminant feed to insect farming results in a reduction in land use and freshwater eutrophication but also in increases in other potential environmental impacts (for example, global warming).

### 6.2. Economic Sustainability

The economic sustainability of food waste valorization is determined by the capacity of circular systems to generate marketable products while simultaneously reducing waste management costs. Consequently, economic benefits should be evaluated through techno-economic analysis (TEA), cost–benefit assessment, and market value generation rather than assumed from resource recovery alone.

A documented example of increased economic sustainability is the valorization of cheese whey. Smithers [163] described the transformation of whey from a disposal burden into a source of whey proteins, lactose derivatives, galacto-oligosaccharides, bioethanol, and lactic acid. The valorization proceeds through the application of innovative separation technologies, both standalone and in combination, for the cost-effective manufacture of whey-based products. The economic success of the dairy industry’s valorization strategy is reflected in the development of a global whey protein market worth several billion euros annually. The conversion of whey into high-value nutritional ingredients has dramatically reduced disposal costs while creating substantial additional revenue streams for dairy processors.

A representative example is the conversion of food waste into platform chemicals and bio-based products. Bastidas-Oyanedel and Schmidt [164] evaluated a food waste biorefinery processing 50 t/day of food waste and compared several valorization scenarios. Conventional anaerobic digestion generated an economic return of approximately 3 USD/t food waste, whereas integrated biorefineries producing organic acids and bioplastics achieved returns of up to 47 USD/t food waste, corresponding to a more than 15-fold increase in value generation. The production of polylactic acid exhibited a return on investment (ROI) of 98% and a payback period of 7.8 years, while butyric acid production achieved a 74% ROI with a payback period of 9.1 years.

The valorization of food waste through insect bioconversion also provides compelling economic evidence. Beesigamukama et al. [165] demonstrated that the production of black soldier fly larvae from organic waste can generate both insect protein and frass fertilizer. The study reported that 1 ton of dried larvae, valued at approximately USD 900, simultaneously generated 10–34 tons of frass fertilizer with an estimated value between USD 3,000 and USD 10,200. When farmers utilized the frass fertilizer directly, net income increased by 30–232%, while maize production fertilized with frass generated 29–44% higher profits than conventional organic fertilization systems.

### 6.3. Social Sustainability

The social benefits of food waste valorization are more difficult to quantify than economic outcomes, yet several studies have demonstrated measurable contributions to food security, resource accessibility, and rural development.

One of the most compelling examples concerns the utilization of food waste for animal feed production. Tchonkouang et al. [166] estimated that recovering and valorizing food waste can contribute to food availability by redirecting nutrients and biomass back into the food system. The authors highlighted a case study showing that avoidable food waste generated in Australia could theoretically provide 1.8 trillion calories, sufficient to feed approximately 921,000 people for one year. Although this represents a theoretical scenario, it illustrates the magnitude of the food security benefits associated with waste prevention and valorization.

The social benefits extend beyond food access. Food redistribution systems create employment opportunities in food collection, logistics, storage, and community services while also strengthening local support networks. Consequently, food rescue is increasingly recognized as a high-priority valorization strategy within the food waste hierarchy because it simultaneously addresses food insecurity, social inequality, and resource inefficiency. One of the most documented examples is the work of Feeding America, the largest food rescue network in the United States. Food recovery systems can redirect substantial quantities of edible surplus food toward vulnerable populations while reducing disposal costs and environmental burdens. More recently, Feeding America reported the redistribution of approximately 5.3 billion meals in 2023 through its network of more than 200 food banks [167]. This demonstrates the enormous social value that can be generated by recovering food that would otherwise be discarded.

### 6.4. Sustainability Assessment Methodologies for Agri-Food Waste Valorization Systems

The evaluation of agri-food waste valorization systems requires a multidimensional assessment framework capable of simultaneously addressing environmental, economic, social, technological, and circularity aspects. Although technical feasibility has traditionally been the focus of food waste valorization research, recent studies increasingly recognize that sustainable implementation requires a broader perspective that integrates environmental impacts, economic viability, social implications, technological maturity, and circular economy performance [168] (Table 5).

#### 6.4.1. Life Cycle Assessment (LCA)

LCA is currently the most widely applied methodology for evaluating the environmental sustainability of agri-food systems and food waste valorization pathways [104]. Standardized through ISO 14040 and ISO 14044, LCA assesses environmental impacts throughout the entire life cycle of a product or process, from raw material acquisition to end-of-life management. It enables the identification of environmental hotspots and supports eco-design and process optimization strategies [171].

LCA has become particularly relevant in food waste valorization because recovering waste streams and transforming them into valuable products may generate both environmental benefits and burdens that are not immediately evident. Dominguez Aldama et al. [172] highlighted that food waste valorization systems involve complex multifunctional processes, requiring careful consideration of allocation procedures, system boundaries, and co-product management to ensure robust environmental assessments. The authors further emphasized that inconsistent allocation methods remain one of the major methodological challenges in LCAs of food waste valorization systems.

Recent applications have demonstrated the usefulness of combining LCA with valorization studies. For example, Arias et al. [173] evaluated the recovery of bioactive compounds from orange peels and tomato seeds using different extraction technologies and showed that environmental performance depends strongly on energy consumption, extraction technology, and production scale.

#### 6.4.2. Techno-Economic Analysis (TEA)

This instrument evaluates the economic feasibility of valorization technologies. TEA integrates process engineering data with economic indicators to determine whether a technology can be successfully implemented at an industrial scale. Common indicators include capital expenditure (CAPEX), operational expenditure (OPEX), net present value (NPV), internal rate of return (IRR), ROI, payback period, and minimum selling price (MSP).

The importance of TEA in agri-food waste valorization has been repeatedly emphasized because many technologies that perform well at laboratory scale may prove economically unfeasible when scaled up. Arias et al. [173] combined LCA and TEA to evaluate different extraction technologies for recovering phenolic compounds from orange peels and tomato seeds, demonstrating that environmental and economic performances are often closely interconnected. Similarly, Barrios et al. [174] performed a techno-economic assessment of microwave-assisted protein extraction from brewer’s spent grain, spent coffee grounds, and kale stems, highlighting the importance of production costs and minimum selling prices in determining commercial viability.

In the bioenergy sector, techno-economic analyses have also been applied to biomass gasification systems. Aguado et al. [175] reported that biomass gasification of olive and almond residues for combined heat and power generation achieved payback periods between five and nine years, demonstrating the potential economic viability of waste-to-energy approaches in the agri-food sector.

#### 6.4.3. Social Life Cycle Assessment (S-LCA)

The social dimension of sustainability has historically received less attention than environmental and economic aspects. However, recent studies increasingly recognize that food waste valorization can influence employment, working conditions, food security, local development, and community well-being. Social Life Cycle Assessment, developed under the United Nations Environment Programme/Society of Environmental Toxicology and Chemistry (UNEP/SETAC) framework, aims to evaluate social impacts across the entire life cycle of a product or process. Kameyama et al. [176] observed that social considerations remain underrepresented in food loss and waste research despite their importance for sustainability assessment. Their review identified three major dimensions frequently considered in S-LCA studies: working conditions, community impacts, and food security.

Worker-related indicators commonly include occupational health and safety, wages, working hours, and labor rights, whereas community indicators address local employment, economic development, and stakeholder engagement. Food security indicators focus on improved food availability, food access, and nutritional benefits. Kameyama et al. [176] further highlighted the need to strengthen the integration of S-LCA with the Sustainable Development Goals (SDGs), particularly those related to food security, education, and partnerships.

#### 6.4.4. Technology Readiness Level (TRL) Assessment

Technological sustainability depends not only on process performance but also on the maturity and scalability of the technology. TRL assessment is widely used to evaluate the progression of technologies from basic research (TRL 1) to full commercial deployment (TRL 9). TRL assessment has become particularly important in food waste valorization because many technologies remain confined to laboratory or pilot scales. Recent reviews emphasize that industrial implementation continues to be constrained by scalability issues, process integration challenges, and technological uncertainties [177]. Consequently, TRL evaluation provides a useful framework for identifying technologies that are closest to industrial adoption and those requiring further development.

#### 6.4.5. Circularity Assessment

The increasing adoption of circular economy principles has stimulated the development of methodologies specifically designed to evaluate resource circularity. Traditional sustainability assessments often fail to capture the degree to which materials, nutrients, and resources are retained within production systems.

The Material Circularity Indicator (MCI), developed by the Ellen MacArthur Foundation, is one of the most widely applied circularity metrics. Rocchi et al. [178] adapted the MCI methodology to the olive oil sector and emphasized its usefulness for assessing resource circulation, waste reduction, and regeneration pathways within agri-food systems. Tetteh et al. [179] subsequently compared the Material Circularity Indicator (MCI) with the Circular Flow Index (CFI) and demonstrated that the combination of both indicators provides a more comprehensive assessment of circularity in biological production systems. Importantly, these studies concluded that circularity indicators should not be applied in isolation but rather combined with life cycle-based metrics to avoid misleading conclusions regarding sustainability.

#### 6.4.6. Integrated Sustainability Assessment and Decision Support

Because no single methodology can comprehensively evaluate sustainability, integrated assessment frameworks are increasingly recommended. Life Cycle Sustainability Assessment (LCSA) combines environmental LCA, Life Cycle Costing, and Social Life Cycle Assessment into a unified framework capable of addressing the three pillars of sustainability simultaneously. Stillitano et al. [168] observed that most circular economy studies in the agri-food sector continue to rely primarily on stand-alone environmental LCAs, while social assessments remain largely absent. The authors therefore recommend broader adoption of integrated life cycle methodologies to better capture the complexity of circular agri-food systems.

In parallel, Multi-Criteria Decision-Making (MCDM) approaches have emerged as valuable decision-support tools. Zhang and Zhang [110] combined SWARA (Step-Wise Weight Assessment Ratio Analysis), DEMATEL-ISM (Decision-Making Trial and Evaluation Laboratory-Interpretive Structural Modeling), and Quality Function Deployment methods to identify the most critical barriers and solutions for agri-food waste valorization, demonstrating the usefulness of MCDM techniques for prioritizing interventions under conditions of uncertainty. SWARA is a method used to calculate the relative weights of various criteria/factors. DEMATEL is a quantitative method used for complicated causal relationships. ISM is a qualitative, structured modeling technique that transforms scattered ideas into a hierarchical structural model.

## 7. Current Challenges and Future Development Roadmap for Food Waste Valorization

Despite remarkable advances in food waste valorization technologies over the past decade, their transition from laboratory-scale demonstrations to economically viable and fully integrated industrial biorefineries remains a major challenge. Although numerous studies have demonstrated the technical feasibility of converting agri-food waste and by-products into bioactive compounds, functional ingredients, biomaterials, biofuels, and platform chemicals, widespread commercialization is still constrained by several scientific, technological, economic, and regulatory barriers. These limitations are particularly evident in integrated biorefinery systems, where multiple processing steps, conversion technologies, and product streams must be efficiently coordinated to maximize resource utilization and economic performance. Feedstock heterogeneity, seasonal variability, process scalability, high capital and operating costs, regulatory uncertainty, and market acceptance remain among the most frequently reported obstacles. Addressing these challenges will require not only technological improvements but also the development of next-generation biorefineries integrating green processing technologies, digitalization, artificial intelligence, synthetic biology, metabolic engineering, and carbon-neutral process design. Consequently, the future roadmap for food waste valorization should focus on creating intelligent, flexible, and sustainable biorefinery systems capable of supporting the transition toward a resilient circular bioeconomy.

### 7.1. Feedstock Heterogeneity

Agri-food waste is intrinsically heterogeneous because it includes materials with different moisture contents, chemical compositions, biodegradability, particle sizes, and contamination risks. Patinha Caldeira et al. [180] specifically identified feedstock security as a key unresolved issue for food waste biorefineries. Food waste composition also depends on whether the stream derives from manufacturing, retail, catering, households, or mixed municipal sources, making classification and treatment selection difficult [181]. This variability directly affects process standardization. Some authors reviewed global food waste valorization options and emphasized that different food waste streams require different valorization pathways because their composition and suitability for conversion technologies vary substantially [14]. For biorefineries, such variability can influence extraction yields, fermentation efficiency, downstream purification, and final product consistency [180]. Seasonality is another major limitation. Many residues, such as olive pomace, grape marc, tomato pomace, citrus peels, and fruit-processing by-products, are generated during short processing periods rather than continuously throughout the year. Ensuring stable feedstock supply is still a critical requirement for moving food waste biorefineries beyond laboratory scale. Stabilization, drying, storage, or preservation may therefore be necessary, but these operations increase cost and may reduce environmental benefits.

### 7.2. Economic Feasibility

Although food waste may appear to be a low-cost feedstock, its valorization can be economically constrained by collection, sorting, transport, pretreatment, extraction, purification, and waste handling costs. Roy et al. emphasized that economic, environmental, and social aspects of food waste valorization are still less developed than technological demonstrations [10]. Many food waste valorization pathways are still insufficiently assessed from a techno-economic perspective, even though such assessment is essential for industrial deployment [182]. In general, processes producing only low-value outputs, such as biogas or compost, are less economically attractive than cascade systems that first recover higher-value ingredients, chemicals, or biomaterials and finally obtain energy. Economic feasibility is also strongly affected by scale. Integrated environmental and techno-economic assessments of biorefineries show that performance depends on feedstock cost, process configuration, product portfolio, energy demand, capital investment, and market price of recovered products [182]. The commercial-scale operation of biorefineries affects the price of feedstock due to their increased demand. This means that the size of the biorefinery is a key factor affecting the cost-effectiveness of the biorefinery together with the selling price of biorefinery products [183,184]. Therefore, techno-economic analysis should be included before scale-up, rather than after laboratory optimization.

### 7.3. Regulatory Issues and Consumer Acceptance

Regulatory barriers are especially critical when recovered compounds are intended for food, feed, nutraceutical, or cosmetic applications. Waste-derived food ingredients must comply with safety requirements related to microbiological hazards, heavy metals, pesticide residues, allergens, mycotoxins, and process contaminants. Food safety assessment in Europe relies on analytical control and metrological reliability for contaminants such as mycotoxins, emerging contaminants, and process contaminants [185]. Another difficulty is the legal and conceptual definition of food waste, by-products, and upcycled ingredients. Furthermore, definitions of food waste differ among FAO (Food and Agriculture Organization of the United Nations), FUSIONS (project funded by the European Commission Framework Programme 7, named Food Use for Social Innovation by Optimizing Waste Prevention Strategies), and WRAP (the UK Waste and Resources Action Programme), particularly regarding edible and inedible fractions and alternative uses such as animal feed [186]. This ambiguity can complicate regulatory classification and market authorization. Generally, countries have not developed a specific regulatory regime for upcycled foods [187]. This means that upcycled foods must primarily comply with general food safety and food labeling requirements. However, some countries have developed a novel food regulatory regime that could also be applied to upcycled foods. Among them, we can include the EU, China, Canada, Brazil, India, Israel, Australia and New Zealand. As an example, in the EU, an upcycled food can be considered a novel food if it “was not used for human consumption to a significant degree within the Union before 15 May 1997” and if it falls into one of the categories identified by the Regulation EU 2015/2283 [188]. In the US, an upcycled food can be regulated through the US food additive approval procedure unless it is a GRAS product or otherwise exempt under relevant Federal law [189].

Consumer acceptance is also a major barrier for waste-derived food ingredients. Moshtaghian et al. [190] reported that upcycled foods face challenges related to definition, inclusion in the food waste hierarchy, and public acceptability. They highlighted the key role of sociodemographic characteristics of consumers, beliefs, and food quality. In this light, communicating the positive aspects of these foods, such as their nature as healthy, nutritious, and environmentally friendly foods, can increase their willingness to purchase, pay for, and consume them [191]. A focus group study was recently organized to give an insight into perception, acceptance, and willingness to buy for foods derived from by-products among a population (18–60 years old range) of four European countries (Italy, Germany, Romania, Norway) [192]. The results showed a high degree of acceptability of these products in all four studied countries, mainly driven by health benefits and contribution to food waste reduction. Differences emerged among participants of various nationalities. For example, participants declared their preference for waste-derived foods belonging to categories such as bakery (except for Norwegians), meat (except for Italians), fruit juices and biscuits (except for Germans), and protein bars (only Norwegian and Romanian people). However, acceptance was given only if the sensory characteristics of new foods were like those of the conventional products. Participants were afraid of possible food allergies and intolerances and concentrations of food contaminants.

### 7.4. Technological Limitations and Scalability

Many valorization technologies, including green extraction, fermentation, enzymatic hydrolysis, anaerobic digestion, and thermochemical conversion, have been successfully demonstrated at laboratory or pilot scale, but industrial scalability and widespread commercial implementation remain limited. Patinha Caldeira et al. explicitly concluded that numerous food waste biorefinery pathways are still mainly developed at a laboratory scale and require assessment of upscaled performance [180].

Scale-up introduces technical challenges that are often not visible in laboratory experiments. These include heat and mass transfer limitations, solvent recovery, process control, microbial contamination, product purification, and long-term operational stability. Integrated biorefineries are even more complex because inefficiency in one unit operation can affect the whole cascade. One of the principal challenges concerns the scale-up of extraction technologies used for recovering bioactive compounds from food waste.

Ultrasound-assisted extraction, microwave-assisted extraction, supercritical fluid extraction, and pulsed electric field technologies frequently achieve high extraction yields under laboratory conditions; however, maintaining process efficiency, energy performance, and solvent recovery at an industrial scale remains difficult. The transition from batch laboratory systems to continuous industrial operations often introduces limitations associated with heat transfer, mass transfer, process control, and equipment design. Biological conversion processes face similar constraints. Fermentation-based technologies are highly sensitive to substrate composition, contamination risks, oxygen transfer limitations, and microbial stability. Furthermore, inhibitory compounds naturally present in some waste streams can negatively affect microbial growth and product yields, requiring additional pretreatment steps that increase process complexity and costs.

Another important limitation is the relatively low technological readiness level (TRL) of many valorization pathways. Patinha Caldeira et al. [180] observed that a large proportion of published studies focus on proof-of-concept demonstrations without addressing long-term operational stability, process robustness, industrial integration, or market deployment. Consequently, many technologies perform well under controlled laboratory conditions but encounter difficulties when subjected to industrial throughput, continuous operation, and variable feedstock supply.

Pilot-scale studies illustrate this challenge. In their pilot-scale investigation of food waste valorization into bioethanol, Passadis et al. demonstrated the technical feasibility of producing ethanol from source-separated food waste, but they also reported that enzyme consumption and energy requirements remained major cost drivers affecting commercial viability. The authors concluded that further optimization of the entire value chain is required before industrial implementation can be achieved [193].

Roy et al. [10] highlighted that many studies report product yields and technical efficiencies but fail to include environmental assessments, techno-economic analyses, or social sustainability indicators, making it difficult to identify the most sustainable pathways for industrial development.

### 7.5. Commercial Barriers

The main commercial barrier to upcycled food and food ingredient diffusion is represented by the limited space that these products occupy on the shelves of retailers. Obtaining larger shelf space for these products in stores is essential to increasing visibility, consumer adoption, and ultimately, the commercial viability of recycled foods. This decision, however, rests with retail category managers, who oversee sourcing and decide on the purchase of products that align with their organization’s strategic objectives [194]. The literature on category managers’ decision-making process for the approval of new food products is still rather limited. According to a 2018 study [195], the high level of autonomy of retail managers implies a high variability in key factors they adopt in the product selection process. However, the factors that positively affect the decision to stock a product include the presence of customer demand for that good, the existence of marketing support, and the provision of a financial return for the supermarket [196]. Price could represent an important barrier because upcycled foods and ingredients are niche products with a higher price than equivalent conventional products [194]. Another important commercial barrier is represented by the relative limited diffusion of unified certification frameworks and labeling frameworks able to ensure transparency and build trust. In fact, the literature suggested that the willingness to pay (WTP) for upcycled products increases when effective nutritional and environmental information is provided to the consumer. However, the type of information supplied affects WTP in a different way depending on the cluster category the consumer belongs to. A case study investigated by Nguyen et al. [197] concerned the willingness of Vietnamese consumers to pay a premium price for biscuits made with brewer’s spent grain with respect to conventional biscuits. The price of a 200 g pack of biscuits ranged from VND (Vietnamese dong) 25,000 to VND 70,000. They individuated two consumer clusters: cluster 1, the so-called “Environmentalist”, who exhibited the highest preferences for sustainability information; and cluster 2, named “Nutritionist”, who demonstrated notable preferences for fiber content. Environmentalist consumers declared a much higher willingness to pay for biscuits with sustainability information (+40,660 VND/pack) while nutritionist consumers declared their availability to pay a much higher price for biscuits with high fiber content (+34,380 VND/pack).

### 7.6. Future Development Roadmap

Although remarkable progress has been achieved in the valorization of food waste into value-added products, the transition from laboratory-scale research to economically viable and industrially implemented biorefinery systems remains a major challenge. Future developments should therefore move beyond the optimization of individual technologies and focus on the design of integrated, intelligent, and circular valorization platforms capable of maximizing resource efficiency while minimizing environmental impacts. Biorefineries will have to be designed as flexible, modular, and cascade-based systems. In cascading biorefineries, food waste is fractionated according to the economic value of its constituents. Instead of directing biomass toward a single end-product, future biorefineries should sequentially recover high-value compounds, including polyphenols, carotenoids, proteins, peptides, dietary fibers, pectin, and essential oils, before converting the remaining biomass into biofuels, biomaterials, platform chemicals, fertilizers, and biochar through biological and thermochemical processes. Such cascade approaches maximize biomass utilization while improving the overall profitability of the biorefinery. Priority should be given to mild and food-grade technologies for recovering high-value compounds, followed by secondary conversion of residual biomass into energy, fertilizers, environmental materials, or bulk chemicals [18].

Future research should increasingly focus on green extraction technologies capable of improving recovery yields while reducing environmental impacts. Sustainable extraction approaches such as ultrasound-assisted extraction, microwave-assisted extraction, pressurized liquid extraction, supercritical fluid extraction, pulsed electric field-assisted extraction, and high-pressure extraction have demonstrated considerable potential for recovering bioactive compounds from agri-food residues [145]. Similarly, emerging technologies including natural deep eutectic solvents, spray drying, freeze-drying, nanoencapsulation, and colloidal delivery systems have been identified as promising tools for enhancing the recovery, stabilization, and functionality of bioactive ingredients derived from agri-food by-products [16]. For these reasons, another important research direction is the integration of multiple green extraction technologies. Hybrid extraction processes combining them are expected to significantly improve extraction yields while reducing solvent consumption, processing time, and energy requirements. However, systematic studies are still required to identify optimal combinations of technologies for different waste streams and target compounds.

Future studies should also prioritize process intensification and continuous processing. Most current extraction methods have been optimized under batch laboratory conditions, whereas industrial implementation requires continuous-flow systems characterized by high productivity, reproducibility, and reduced operational costs. The design of modular and flexible biorefinery platforms capable of processing heterogeneous feedstocks throughout the year will represent a key step toward commercialization. Another promising research area concerns the development of sustainable solvents and extraction media. Although deep eutectic solvents (DESs), natural deep eutectic solvents (NADESs), and pressurized NADESs have attracted considerable attention, further studies are needed to evaluate their toxicity, biodegradability, solvent recovery, recyclability, and regulatory acceptance before large-scale industrial adoption. Likewise, membrane-based purification systems should be further optimized to reduce fouling and improve long-term operational stability.

The development of functional foods, nutraceuticals, and health-promoting ingredients derived from food waste is expected to become a major research area in the coming years. Numerous agri-food by-products contain substantial quantities of polyphenols, carotenoids, dietary fibers, proteins, vitamins, and others, often at concentrations equal to or higher than those found in edible plant tissues [18,145]. Consequently, future studies should not only optimize extraction processes but also investigate bioavailability, stability, encapsulation strategies, and incorporation into food matrices [145].

Innovative manufacturing technologies may further expand opportunities for food waste valorization. Three-dimensional food printing has recently emerged as a promising strategy for transforming food-processing residues into customized foods and biodegradable materials. The technology offers high levels of customization, supports zero-waste manufacturing concepts, and enables the incorporation of waste-derived nutrients and functional ingredients into novel food formulations [19].

The digital transformation of biorefineries is expected to become one of the main drivers of innovation during the coming decade. Artificial intelligence, machine learning, digital twins, Internet of Things (IoT) technologies, advanced sensors, and big data analytics will increasingly support process optimization, predictive maintenance, quality control, and real-time decision-making [16]. Digital twins will enable virtual simulation of extraction, fermentation, and thermochemical conversion processes, thereby reduce scale-up risks and facilitating process optimization before industrial implementation. Similarly, machine learning algorithms will allow prediction of extraction yields, optimization of operating conditions, and intelligent selection of processing routes according to biomass characteristics.

Another transformative research direction concerns the integration of synthetic biology, metabolic engineering, and precision fermentation into food waste biorefineries. Recent advances in genome editing, systems biology, and synthetic biology enable the rational design of microbial cell factories capable of converting heterogeneous food waste streams into high-value products with unprecedented efficiency and selectivity. Through metabolic pathway engineering, microorganisms such as *Escherichia coli, Saccharomyces cerevisiae, Yarrowia lipolytica, Bacillus subtilis*, and filamentous fungi can be engineered to overproduce several compounds of interest (organic acids, amino acids, carotenoids, enzymes, vitamins, and bioactive peptides) using food waste hydrolysates as renewable carbon sources. The result will be the development of robust microbial platforms with improved substrate utilization, inhibitor tolerance, and product yields. *Yarrowia lipolytica* has emerged as one of the most promising microbial chassis for the valorization of agri-food residues owing to its broad substrate spectrum, oleaginous metabolism, and extensive synthetic biology toolbox [198,199]. Future biorefineries are also expected to exploit synthetic microbial consortia, in which multiple engineered microorganisms cooperate to sequentially convert complex biomass into a portfolio of valuable products. Such division-of-labor strategies can overcome many of the limitations associated with single-strain fermentations and enable the simultaneous production of multiple biochemicals.

Another major objective for future research is the development of carbon-neutral or even carbon-negative biorefineries, in which carbon is continuously recycled rather than released into the atmosphere. Future facilities should integrate renewable electricity, carbon capture and utilization, anaerobic digestion, thermochemical conversion, nutrient recovery, and biochar production within closed-loop processing systems. Carbon dioxide generated during fermentation, anaerobic digestion, or gasification could be captured and reused for microbial fermentation, algal cultivation, or chemical synthesis, thereby generating additional value streams while reducing greenhouse gas emissions. Likewise, biochar produced through pyrolysis or hydrothermal carbonization may contribute to long-term carbon sequestration while simultaneously improving soil fertility and agricultural productivity. The concept of net-zero biorefineries extends beyond greenhouse gas mitigation and aims to optimize carbon, water, energy, and nutrient cycles simultaneously. Accordingly, future industrial facilities should increasingly adopt principles of industrial symbiosis, in which waste streams generated by one process become feedstocks for another, maximizing overall resource efficiency and minimizing environmental burdens. Such integrated systems are fully aligned with the biorefinery classification and cascading concepts proposed within the IEA Bioenergy Task 42 framework and subsequent developments in circular bioeconomy research [200]. The convergence of synthetic biology, artificial intelligence, digital twins, advanced process control, and renewable energy integration will likely define the next generation of smart carbon-neutral biorefineries. These facilities will combine predictive process optimization, real-time monitoring, engineered microbial platforms, and circular resource management to manufacture food ingredients, biomaterials, chemicals, and bioenergy with minimal waste generation and reduced greenhouse gas emissions. Such multidisciplinary approaches represent one of the most promising pathways toward resilient, climate-neutral, and economically sustainable circular bioeconomy systems.

Future studies should also place greater emphasis on integrated sustainability assessment frameworks. Although numerous valorization technologies have demonstrated technical feasibility, relatively few studies have simultaneously evaluated their environmental, economic, and social impacts. Consequently, Life Cycle Assessment (LCA), Techno-Economic Analysis (TEA), Social Life Cycle Assessment (S-LCA), and Life Cycle Sustainability Assessment (LCSA) should become standard components of future research to identify truly sustainable and scalable valorization pathways [65].

The transition toward sustainable food systems will also require stronger collaboration among researchers, industry, policymakers, and consumers. Therefore, future policy frameworks should encourage investment in circular bioeconomy initiatives, support technological innovation, and facilitate the development of markets for upcycled products [201]. Regulatory harmonization will play a crucial role in accelerating industrial implementation. Clear regulatory frameworks governing the safety, quality, traceability, and commercialization of waste-derived ingredients remain limited in many countries. Future legislation should support the safe use of food waste-derived ingredients while promoting consumer confidence through transparent certification schemes, standardized quality criteria, and digital traceability systems.

## 8. Conclusions

Food waste valorization has evolved from a waste management strategy into a key pillar of the circular bioeconomy, offering opportunities to recover valuable compounds while simultaneously reducing environmental impacts and improving resource efficiency. Recent advances in green extraction technologies, biochemical and thermochemical conversion, biorefinery concepts, and functional food applications demonstrate that agri-food waste can serve as a renewable feedstock to produce bioactive compounds, functional ingredients, biomaterials, biofuels, and other value-added products. Nevertheless, the widespread industrial implementation of these technologies still requires overcoming important challenges related to feedstock variability, process integration, economic competitiveness, regulatory harmonization, and market acceptance. Future progress will depend on the development of intelligent cascading biorefineries integrating green processing technologies with artificial intelligence, digital twins, synthetic biology, metabolic engineering, and comprehensive sustainability assessment tools such as LCA and TEA. Future interdisciplinary research integrating biotechnology, food science, environmental engineering, and circular economy principles will be essential for unlocking the full potential of food waste valorization and achieving global sustainability goals.

## Figures and Tables

**Table 1:** Food waste sources and composition.

Waste Sectors	Reported Waste Streams	Reported Compositions	Examples of References
Cereals and pulses	Germ, bran, husks, stems, leaves, rice bran, rice husk	Vitamin E, phytates, phenolics, insoluble dietary fiber	[17]
Fruit and vegetable processing	Peels, skins, pomace, pulp, seeds, stems	Water, soluble carbohydrates, fiber, minerals, vitamins, polyphenols, carotenoids, enzymes	[17,18,19]
Wine/grape industry	Grape pomace, skins, seeds	Polyphenols, proanthocyanins, resveratrol derivatives, antioxidant compounds	[20,21]
Olive oil sector	Olive-oil processing residues, olive leaves	Phenolic compounds, including oleuropein	[20]
Dairy sector	Whey, milk-processing wastewater	Lactose/carbohydrates, proteins, lipids, minerals, organic matter	[17,22]
Meat, poultry, eggs	Feathers, skin, bones, blood, fat, soft tissues	Proteins, fats, organic matter, collagen/keratin-rich fractions	[17]
Seafood and aquatic products	Shrimp shells, crab shells, prawn waste, fish scales, fish bones	Proteins, chitin, calcium carbonate, organic and inorganic fractions	[17,19]
Mixed agri-food by-products	Shells, seeds, effluents, fibrous materials, pulp, leaves, roots	Phenolics, fibers, proteins, functional lipids, minerals, pigments	[16]
Biopolymer-oriented waste streams	Plant, animal, microbial wastes/by-products	Lignin, cellulose, pectin, starch, chitosan, PHA, PLA	[23,24]

**Table 2:** Representative methodologies for agri-food waste valorization, products obtained, and key supporting references.

Valorization Methodologies	Main Technologies	Representative Feedstocks	Main Products Obtained	Examples of References
Recovery of bioactive compounds	UAE, MAE, SFE, SWE, PEF, HHP, EAE, DES/NADES	Fruit peels, grape pomace, tomato waste, olive residues, citrus by-products	Polyphenols, flavonoids, carotenoids, anthocyanins, essential oils	[32,57,60]
Functional food ingredients	Extraction, membrane filtration, drying, fractionation	Apple pomace, citrus peels, cereal bran, brewer’s spent grain	Dietary fibers, pectin, proteins, natural colorants	[57,60]
Nutraceutical production	Green extraction, encapsulation, fermentation	Grape pomace, olive leaves, fruit peels, berry residues	Antioxidant extracts, polyphenol concentrates, nutraceutical powders	[20,57]
Natural food additives	Extraction and purification	Citrus peels, tomato waste, grape pomace	Pectin, pigments, antioxidants, preservatives	[57,60]
Animal feed production	Drying, fermentation, bioconversion	Fruit pomace, vegetable residues, cereal by-products	Feed ingredients, protein-rich feed, silage	[57,60]
Single-cell protein production	Yeast and fungal fermentation	Fruit wastes, brewer’s spent grain, food waste hydrolysates, whey	Microbial protein biomass	[57,65,87]
Bioactive peptides and amino acids	LAB fermentation, Bacillus fermentation, solid-state fermentation (SSF)	Whey, soybean residues, coffee grounds, fish waste	Bioactive peptides, GABA, amino acids	[68,70]
Platform chemicals	Fermentation, catalytic conversion	Carbohydrate-rich food waste, fruit residues	Lactic acid, succinic acid, citric acid, furfural, HMF	[57,60]
Biopolymers and biomaterials	Fermentation, microbial synthesis, polymer extraction	Food waste hydrolysates, fruit and vegetable residues	PLA, PHA, cellulose materials, biodegradable films	[57,88]
Biofertilizers and bio-stimulants	Composting, vermicomposting, extraction, fermentation	Crop residues, food-processing waste, digestate	Compost, biofertilizers, bio-stimulants	[89,90]
Insect bioconversion	BSFL	Household food waste, fruit and vegetable waste	Insect protein, insect oil, frass fertilizer	[81,82,83]
Fermentation-based valorization	SSF, Submerged Fermentation (SmF), mixed-culture fermentation	Fruit pomace, cereal residues, agri-industrial wastes	Organic acids, enzymes, SCP, microbial metabolites	[57,60]
Anaerobic digestion	AD, co-digestion	Food waste, dairy waste, crop residues, agri-industrial effluents	Biogas, biomethane, digestate	[22,56]
Thermochemical conversion	Pyrolysis, gasification, HTC, hydrothermal liquefaction (HTL)	Lignocellulosic residues, food waste, digestate	Biochar, bio-oil, syngas, hydrochar	[91,92]
Integrated biorefineries	Cascade extraction + biochemical + thermochemical conversion	Apple pomace, tomato waste, grape pomace, olive pomace	Bio-actives, chemicals, materials, fertilizers, bioenergy	[56,91]
Digital and AI-assisted biorefineries	Artificial intelligence, machine learning, digital twins	Multiple waste streams	Process optimization, yield prediction, decision support	[57]
Nanotechnology-assisted valorization	Nano-encapsulation, nano-emulsions, nanocarriers	Polyphenol-rich extracts, carotenoid-rich residues	Functional foods, nutraceuticals, delivery systems	[93]

**Table 3:** Representative applications of green and emerging extraction technologies to food waste and by-products.

Technology	Food Waste/By-Product	Target Fraction	Extraction Medium/Pretreatment	Representative Application Conditions	Main Result/Process Note	Reference
UAE	Black and purple rice bran	Phenolic compounds and anthocyanins	Aqueous ethanol; milled bran	Ultrasonic treatment at 40 kHz; solvent composition, temperature and treatment time optimized by comparative extraction trials.	UAE increased the recovery of bran phenolics and anthocyanins while retaining antioxidant activity.	[94]
UAE	Onion solid waste	Quercetin, flavonoids and pigments	90% (*w*/*v*) aqueous glycerol	Probe/bath ultrasonication combined with a glycerol-water solvent; temperature, glycerol concentration and time optimized.	A food-compatible glycerol-rich solvent gave efficient recovery without conventional volatile organic solvents.	[95]
MAE	Industrial potato peel	Chlorogenic acid and total phenolics	Aqueous ethanol	Microwave power, ethanol concentration, liquid-to-solid ratio and extraction time were optimized by response-surface methodology; extraction was completed within minutes.	Rapid heating improved phenolic recovery compared with conventional solvent extraction.	[96]
MAE	Spent espresso coffee grounds	Chlorogenic acids, caffeine and antioxidant compounds	Ethanol-water mixtures	Microwave power, solvent composition, solvent-to-solid ratio and irradiation time are optimized; short irradiation is followed by filtration.	MAE reduced extraction time and solvent demand while producing antioxidant-rich extracts.	[97]
PLE	Peach pomace	Phenolic compounds followed by pectin	Aqueous ethanol, then acidified water	Sequential pressurized extraction: hydroethanolic stage for phenolics followed by hot acidified-water extraction for pectin; solvent is held liquid under pressure.	Enabled cascade fractionation into a phenolic-rich extract and a pectin-rich fraction.	[98]
PLE	Olive pomace	Hydroxytyrosol derivatives and other phenolics	Ethanol-water mixtures	Central-composite design varying temperature and ethanol percentage under pressurized conditions; short static extraction cycles.	Selectivity and phenolic profile changed markedly with temperature and solvent composition.	[99]
ASE	Date-palm seeds	Phenolics and antioxidant compounds	Deep eutectic solvent/water in ASE cells	ASE is combined with a DES; temperature, DES composition, water addition and static time are optimized.	The synergistic ASE-DES approach enhanced recovery from a hard seed matrix.	[100]
ASE	Potato-processing by-products	Phenolic compounds	Water, ethanol and aqueous ethanol	Automated pressurized cells; elevated temperature, approximately 10 MPa, and repeated short static cycles.	ASE shortened processing time and reduced solvent use compared with prolonged conventional extraction.	[101]
SFE	Coffee silverskin	Lipid fraction, tocopherols and diterpenes	Supercritical CO_2_	CO_2_ pressure and temperature varied in the supercritical region; dynamic extraction after a static equilibration stage.	Produced a solvent-free semi-solid fat and demonstrated scale-up potential for a roasting by-product.	[102]
SFE	Shrimp-processing waste	Astaxanthin-rich carotenoid fraction	Supercritical CO_2_ with ethanol modifier	Pressure, temperature, CO_2_ flow and co-solvent percentage were optimized; dried and milled shell waste was used as feed.	Co-solvent addition improved recovery of the relatively polar carotenoid fraction.	[103]
SWE	Onion-skin waste	Quercetin and phenolic compounds	Pressurized water	Water maintained in the liquid state at 100–200 °C; temperature and residence time varied; no organic solvent.	Higher temperature lowered water polarity and accelerated phenolic release, although severe conditions increased degradation risk.	[104]
SWE	Coffee silverskin	Chlorogenic acids and antioxidant compounds	Pressurized water	Mild hydrothermal treatment at subcritical-water conditions followed by solid–liquid separation; temperature/time selected to recover antioxidants before carbohydrate valorization.	Supported a cascade biorefinery combining antioxidant recovery and subsequent sugar production.	[105]
HC	Pomegranate processing by-products	Ellagitannins, anthocyanins and antioxidant phytocomplexes	Water	Whole non-edible fractions dispersed in water and circulated through a cavitation reactor; sequential fractions collected during processing.	Generated water-based antioxidant extracts without organic solvents and were suitable for comparatively large liquid volumes.	[106]
HC	Waste orange peel	Pectin, flavanones, hydroxycinnamic acids and terpenes	Tap water	Several kilograms of peel processed in >100 L water in a Venturi-type reactor; valuable compounds released rapidly during recirculation.	Demonstrated integral, solvent-free valorization at a pre-industrial scale.	[107]
CPAE	De-oiled rice bran	Ferulic, sinapic, vanillic and chlorogenic acids	Atmospheric or vacuum plasma pretreatment, followed by solvent extraction	Plasma type and exposure duration varied before extraction; low-temperature treatment generated surface fissures in bran particles.	Plasma pretreatment increased total and individual phenolic recovery and improved in vitro bioactivity indicators.	[108]
CPAE	De-oiled corn bran	p-Coumaric, ferulic and sinapic acids	Atmospheric or vacuum plasma pretreatment, followed by solvent extraction	The same atmospheric/vacuum plasma configurations were compared on corn bran before conventional extraction.	Atmospheric plasma was particularly effective for improving extractability from the corn-bran matrix.	[108]
OHAE	Red grape pomace	Polyphenols and anthocyanins	Water containing 0–50% ethanol	Pulsed ohmic pretreatment at 100–800 V cm^−1^, followed by diffusion extraction; the best reported combination included 400 V cm^−1^, 30% ethanol, 50 °C and 60 min.	Combined electroporation and rapid volumetric heating increased polyphenol recovery relative to untreated controls.	[109]
OHAE	Grape pomace	Phenolic compounds and bioaccessible antioxidants	Food-grade hydroethanolic medium	Moderate-electric-field ohmic heating is applied during extraction; electric field, temperature and solvent composition are controlled.	Improved extraction kinetics and produced extracts were subsequently assessed for composition, bioactivity and bioaccessibility.	[110]
Membrane separation technologies	Wet olive-pomace aqueous extract	Hydroxytyrosol and related biophenols	UAE water extract used as membrane feed	Sequential ultrafiltration and nanofiltration; commercial membranes evaluated at different transmembrane pressures and cross-flow conditions.	UF purified biophenols into the permeate; NF concentrated the phenolic fraction.	[111]
Membrane separation technologies	Red wine lees	Polyphenols and organic acids	Clarified liquid lees extract	Sequential membrane filtration, including UF/NF, followed by concentration and spray drying.	Produced a concentrated antioxidant powder while separating lower-molecular-mass acids and phenolics from suspended matter.	[112]
PEF	Red grape pomace	Anthocyanins and total phenolics	PEF pretreatment followed by aqueous/hydroethanolic diffusion	Electric field strength of 0.5–5 kV cm^−1^ and specific energy of 1–20 kJ kg^−1^ were examined, with subsequent solvent extraction under mild temperature.	Electroporation improved solvent accessibility and phenolic recovery with limited thermal load.	[113]
PEF	Fermented grape pomace	Selective polyphenol fraction	Densification plus PEF, followed by diffusion extraction	Mechanical densification combined with PEF before solvent diffusion; electric treatment selected to maximize tissue disintegration.	The combined treatment improved selective polyphenol recovery and reduced solvent contact time.	[114]
DES	Aglianico grape pomace	Anthocyanins, flavan-3-ols, flavonols and stilbenes	Choline chloride-oxalic acid (1:1 molar ratio)	DES extraction at 40 °C; water content and extraction time evaluated against methanol and acetone controls.	The DES produced a high total phenolic recovery and a broad phenolic profile.	[115]
DES	Date-palm seeds	Phenolics and antioxidant compounds	DES combined with accelerated solvent extraction	Hydrogen-bond donor/acceptor composition, added water, ASE temperature and static time optimized.	Demonstrated DES suitability for extracting antioxidants from a dense, lignocellulosic seed by-product.	[100]
NADES	Winemaking by-products	Phenolic compounds	Choline chloride-urea NADES added to pressurized water	Approximately 30% NADES in the extraction medium and 100 °C under pressurized/subcritical-water conditions.	The combined p-NADES process improved phenolic extraction while limiting conventional organic-solvent use.	[116]
NADES	Grape pomace from different cultivars	Anthocyanins and other polyphenols	Metabolite-based NADES with controlled water addition	NADES formulation, water percentage, temperature and extraction time optimized; ultrasound used as an enabling technology.	Performance depended on both NADES composition and grape cultivar, showing compound-class selectivity.	[117]
EAE	Pisco grape pomace	Phenolic compounds	Tannase and cellulase in aqueous medium	Optimized treatment: 0.75 U mL^−1^ tannase, 40 U mL^−1^ cellulase, 20 °C and 15 min before recovery of soluble phenolics.	Cell-wall and tannin hydrolysis increased release of antioxidant phenolics under mild conditions.	[118]
EAE	Red grape pomace	Bound polyphenols	Cellulase, pectinase and hemicellulase, alone or combined	Enzyme type/dose, pH, temperature and hydrolysis time are optimized before separation of the extract.	Enzyme selection changed both total recovery and the profile of individual phenolic compounds.	[119]

**Table 4:** Representative commercial products currently utilizing upcycled ingredients.

Companies	Upcycled Ingredients	Commercial Products	Examples of Reference
Renewal Mill	Okara (soy-milk pulp)	Organic Okara Flour, baking mixes, cookies	[150]
Renewal Mill	Oat milk pulp	Oat protein ingredient	[150]
Renewal Mill	Pineapple juice pulp	Pineapple fiber ingredient	[150]
ReGrained	Brewer’s spent grain	SuperGrain+^®^ flour, snack bars	[151]
Pulp Pantry	Vegetable juice pulp	Vegetable chips	[152]
Rind Snacks	Fruit peels	Dried fruit snacks	[153]
Barnana	Surplus bananas	Banana snacks	[154]
Toast Brewing	Surplus bread	Beer	[155]
EverGrain^®^	Brewer’s spent grain	Protein and fiber ingredients	[156]
Take Two Foods	Upcycled barley	Barley milk beverage	[157]
Matriark Foods	Surplus vegetables	Sauces, soups, culinary bases	[158]
Salt & Straw	Okara, spent grain, cacao pulp, surplus bread	Ice cream products	[159]

**Table 5:** Main methodologies used to assess the sustainability and feasibility of agri-food waste valorization systems.

Typologies	Methodologies	Main Purposes	Typical Indicators	Main Outputs
Environmental	LCA (ISO 14040/14044) [169,170]	Quantify environmental impacts across the entire life cycle of the valorization process	Global warming potential, energy demand, water footprint, eutrophication, acidification, land use, resource depletion	Environmental hotspots, carbon footprint, environmental performance of alternative valorization pathways
Economic	Techno-Economic Analysis (TEA) and Life Cycle Costing (LCC)	Evaluate economic viability and industrial competitiveness	Capital expenditure (CAPEX), operational expenditure (OPEX), net present value (NPV), internal rate of return (IRR), return on investment (ROI), payback period, minimum selling price (MSP)	Profitability, investment attractiveness, economic feasibility and cost-effectiveness
Social	Social Life Cycle Assessment (S-LCA)	Assess social impacts on stakeholders throughout the value chain	Employment generation, worker safety, local income creation, food security, stakeholder well-being, community development, consumer acceptance	Social benefits and risks, contribution to local development and societal well-being
Technological	TRL assessment and Process Performance Evaluation	Determine technological maturity, scalability, and operational performance	TRL, process yield, recovery efficiency, conversion efficiency, energy efficiency, operational stability	Technology maturity, scale-up potential, industrial readiness
Circularity	Material Circularity Indicators (MCI) and Circular Economy Metrics	Measure resource efficiency and circularity performance	Resource recovery rate, waste diversion rate, recycling efficiency, nutrient recovery, biomass utilization efficiency	Degree of circularity and resource recirculation within the system
Integrated Sustainability	Life Cycle Sustainability Assessment (LCSA)	Simultaneously evaluate environmental, economic, and social sustainability	Combined LCA, LCC, and S-LCA indicators	Holistic sustainability performance and trade-off analysis
Decision Support	Multi-Criteria Decision Analysis (MCDA)	Compare alternative valorization scenarios considering multiple sustainability criteria	Weighted environmental, economic, social, and technical indicators	Ranking and selection of the most sustainable valorization pathways

## Data Availability

No new data were created or analyzed in this study. Data sharing is not applicable to this article.

## Abbreviations

The following abbreviations are used in this manuscript:

AD	Anaerobic Digestion
AI	Artificial Intelligence
ASE	Accelerated Solvent Extraction
BSFL	Black Soldier Fly Larvae
BSG	Brewer’s Spent Grain
CAPEX	Capital Expenditure
CFI	Circular Flow Index
CPAE	Cold Plasma-Assisted Extraction
DEMATEL-ISM	Decision-Making Trial and Evaluation Laboratory-Interpretive Structural Modeling
DES	Deep Eutectic Solvents
DT	Digital Twin
EAE	Enzyme-Assisted Extraction
FAO	Food and Agriculture Organisation of the United Nations
FUSIONS	Food Use for Social Innovation by Optimizing Waste Prevention Strategies
GRAS	Generally Recognized As Safe
HC	Hydrodynamic Cavitation
HHP	High Hydrostatic Pressure
HTC	Hydrothermal Carbonization
HTL	Hydrothermal Liquefaction
HTG	Hydrothermal Gasification
IEA	International Energy Agency
IoT	Internet of Things
IRR	Internal Rate of Return
LAB	Lactic Acid Bacteria
LCA	Life Cycle Assessment
LCC	Life Cycle Costing
LCSA	Life Cycle Sustainability Assessment
MAE	Microwave-assisted extraction
MCDA	Multi-Criteria Decision Analysis
MCDM	Multi-Criteria Decision-Making
MCI	Material Circularity Indicators
MF	Microfiltration
ML	Machine Learning
MSP	Minimum Selling Price
NADES	Natural Deep Eutectic Solvents
NF	Nanofiltration
NPV	Net Present Value
OHAE	Ohmic Heating-Assisted Extraction
OPEX	Operational Expenditure
p-NADES	Pressurized Natural Deep Eutectic Solvents
PBL	*Protaetia brevitarsis* larvae
PEF	Pulsed Electric Field
PHAs	Polyhydroxyalkanoates
PLA	Polylactic Acid
PLE	Pressurized Liquid Extraction
PUI	Product Containing Upcycled Ingredients
RO	Reverse Osmosis
ROI	Return on Investment
S-LCA	Social Life Cycle Assessment
SCWG	Supercritical Water Gasification
SDG	Sustainable Development Goal
SFE	Supercritical Fluid Extraction
SmF	Submerged Fermentation
SSF	Solid-State Fermentation
SWARA	Step-Wise Weight Assessment Ratio Analysis
SWE	Subcritical Water Extraction
TEA	Techno-Economic Analysis
TRL	Technology Readiness Level
UAE	Ultrasound-Assisted Extraction
UF	Ultrafiltration
UFA	Upcycled Food Association
UI	Upcycled Ingredient
VND	Vietnamese Dong
WRAP	Waste and Resources Action Programme
WTP	Willingness To Pay
USD	United Stated Dollar
